# The Mechanism of Nitrite Reductase

**DOI:** 10.1002/jcc.70088

**Published:** 2025-03-24

**Authors:** Per E. M. Siegbahn

**Affiliations:** ^1^ Department of Organic Chemistry, Arrhenius Laboratory Stockholm University Stockholm Sweden

**Keywords:** cytochrome c nitrite reductase, DFT, mechanisms, quantum chemistry, redox enzymes

## Abstract

Cytochrome c nitrite reductase (CcNiR) activates nitrite and produces ammonia. It is one of several enzymes that use a redox‐active cofactor to perform its reaction. In this case, the cofactor has a heme with a lysine as the proximal ligand and a charged nearby arginine. The role of a tyrosine, which is also close, has been less clear. There are also four bis‐histidine‐ligated hemes involved in the electron transfers. CcNiR has been studied before, using essentially the same methods as here. However, the mechanism is very complicated, involving six reductions, and quite different results for the mechanism have been obtained here. For example, the tyrosine has here been found to be redox active in the final step when ammonia is produced. Also, the arginine has here been found to stay protonated throughout the mechanism, which is different from what was found in the previous study. The present results are in very good agreement with experimental findings and are, therefore, another case where the methodology has been shown to work very well. Previous examples include Photosystem II and Nitrogenase, normally considered to be the most important enzymes in nature for the development of life.

## Graphical abstract

The mechanism for Cytochrome c nitrite reductase (CcNiR) has been obtained, including all six reduction steps. The protonation of NO was found to be the most difficult step to model.

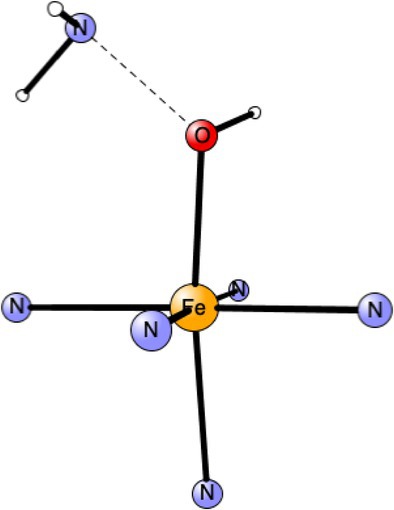

## 1 Introduction

In an effort to model the most important small molecule activations in redox enzymes, the turn has come to cytochrome c nitrite reductase (CcNiR). These studies are made both because of their large chemical interest, but also for testing the methodology. In previous studies, the methodology has been successfully used to determine the mechanism for water oxidation in photosystem II [1, 2], and more recently, for nitrogen fixation in nitrogenase [3, 4], methane and ammonia oxidation in particulate methane monooxygenase (pMMO) [5], and the nitrification in the P460 enzymes hydroxylamine oxidase and cytochrome‐P460 [6]. Earlier work prior to 2014 has been described in a review [7].

CcNiR is an important enzyme in the nitrogen cycle [8, 9]. The reaction carried out is:

$${{\mathrm{NO}}_{2}}^{-}+6\;e^{-}+7\;H^{+}={\mathrm{NH}}_{3}+2\;H_{2}O$$

**FIGURE 1:**
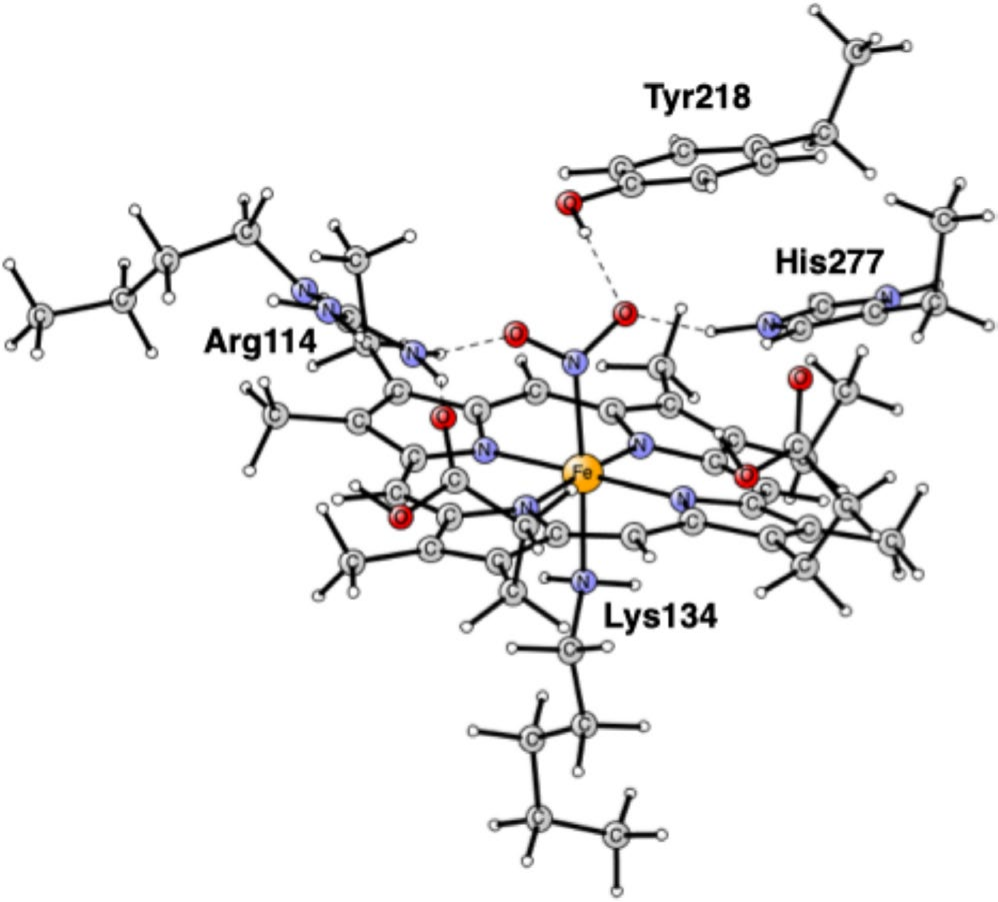
Model of the active site of cytochrome c nitrite reductase (CcNiR) taken from the PDB structure 2E80 [10].

The structure of CcNiR was obtained already in 1999 for *Desulfovibrio vulgaris* [10]. Many other nitrite reductase enzymes have been crystallographically characterized since then. An arrangement of five hemes in the catalytic domain is the same for all of them. Four of them are bis‐histidine ligated and act as electron carriers. The structure of the active site, as modeled here, is shown in Figure 1. There is an unsubstituted heme with an unusual lysine as the proximal ligand. There are three important amino acids in the close neighborhood, all of significant importance for the substrate reaction. Arg114 is charged and in close contact with the substrate. His277 is involved in proton delivery. The role of Tyr218 has been less clear, but it has been suggested that, apart from being involved in proton transfer, it could be redox active forming a tyrosyl radical [10].

The mechanism of CcNiR has been studied by a theoretical modeling in a series of papers [11, 12, 13, 14]. In one of them, all steps of the mechanism have been studied. It was found that a dominating role for proton delivery to the substrate was played by His277, which was expected based on experiments. However, the most interesting findings were a proton donation from Arg114 and a proton delivery role of Tyr218, providing the first theoretical evidence for its role in the overall reaction pathway. A tyrosyl radical never appeared in the suggested mechanism.

In the present paper, the same methodology has been used as in all our recent papers [3, 4, 5, 6]. Detailed comparisons will be made to the previous mechanistic modeling studies of CcNiR, in particular, to the one where all steps were studied [14].

## 2 Methods

Density functional theory has been used with the B3LYP functional [15]. One difference compared to the original functional is that only 15% exact exchange was used instead of 20%. The method has been carefully tested and compared to available experiments for many redox enzymes [7, 16, 17]. Typical errors of 3 kcal/mol and less were generally found. The geometries were optimized using a medium‐size basis set, lacvp*. For the final energies, a large basis set was used (cc‐pvtz(−f)) for the non‐metal atoms, while a lacv3p+ basis was used for iron. For zero‐point effects and determination of transition states, Hessians were computed using B3LYP with lacvp*. The lacvp* and lacv3p basis sets contain a pseudopotential. A dielectric constant of 4.0 was used for the solvation effects when a (H^+^, e^−^) couple was added to the cofactor. For cases where only an electron or a proton was added, a recommended dielectric constant of 20 was instead used [18], but that procedure is less well tested. The D2 approximation was used for the dispersion effects [19]. The Jaguar [20] and Gaussian [21] programs were used.

When redox enzymes are studied, an energy value for the cost of obtaining a (H^+^, e^−^) couple has to be estimated, where one proton comes from the surrounding water medium and the electron comes from the electron donor. The same value as used in the previous study of 369 kcal/mol [14] was used also here. When only a proton was donated to the cofactor, a cost of 180 kcal/mol was used [22].

A cluster modeling of the active site was used [23]. The model is shown in Figure 1. It is essentially the same as the one used in the previous studies [11, 12, 13, 14], which means that the four hemes surrounding the active site were left out. Some atoms are frozen, which is needed in cluster modeling. The atoms frozen are marked with a # in the SI. The only goal with freezing atoms is to keep the structure as close as possible to the X‐ray structure, which can be done in several ways. To achieve this, without disturbing the chemistry, the model must be big enough, and the fixed atoms should be distributed in the periphery of the model.

The number of atoms is about 160. When iron is Fe(II) the spin is zero, and when iron is Fe(III) the spin is one.

When water or ammonia has been released, the binding in the water medium uses an empirical value of 14 kcal/mol, which includes entropy. Based on experience from previous studies, the effect of entropy should be small in the present case. In other studies where molecules like O_2_ or N_2_ are involved, it has been found that entropy effects can be large due to large changes in translational entropy. In those cases, the change in translational entropy has been included, but there are no such cases here.

## 3 Results

The calculations on the mechanism of CcNiR were performed as described in Section 2. The first structure that was optimized is shown in Figure 1. Nitrite is unprotonated and binds with its nitrogen to Fe(II). His277 is protonated as in the previous study [14]. Following the recipe given in Section 2, the proton on His277 is strongly bound by 13.5 kcal/mol, which leads to a charge of +1 for the model at this stage. The spin state is a singlet. There are several important hydrogen bonds. One of the oxygens of the substrate is strongly hydrogen bonded to His277 with a distance of 1.58 Å and a weaker one to Tyr218 with 1.90 Å. The other oxygen is hydrogen bonded to Arg114 with a distance of 1.81 Å. These hydrogen bonds are of significant importance for the first N—O bond cleavage.

In the first step of the mechanism, a proton from the water medium is added to one of the oxygens of the nitrite substrate. The addition of the proton was found to be endergonic by +14.9 kcal/mol. Interestingly, some spins appear in the structure, even though the state is a singlet. The spin on nitrite becomes +0.37 and the spin on iron is −0.42. The structure is shown in Figure S1. At this stage, the addition of an electron is very unfavorable since iron cannot be reduced from Fe(II). Instead, an added electron will end up on the NO_2_H substrate. The (H^+^, e^−^) addition, instead of just a proton, was found to be endergonic by +22.0 kcal/mol.

**FIGURE 2:**
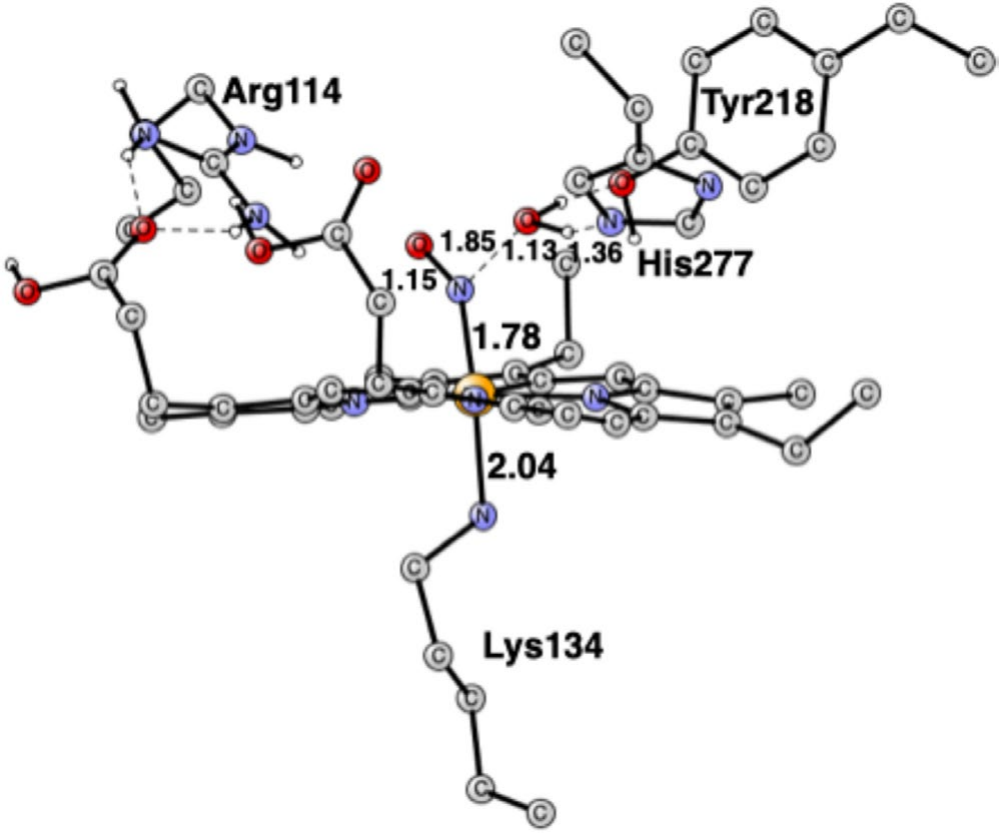
Optimized TS for the cleavage of the first N—O bond. Distances are in Å. The imaginary frequency is 210 cm^−1^.

In the next step, the first N—O bond is cleaved with the TS shown in Figure 2. It should be noted that no reduction is needed so far. The only amino acid directly involved in the N—O cleavage is His277, but there is also a rather weak hydrogen bond to Tyr218, with a distance of 1.80 Å. On the TS structure, it can be seen that the N—O bond is cleaved at a distance of 1.85 Å with a simultaneous proton transfer from His277. The proton is halfway between the substrate and His277, with distances of 1.13 and 1.36 Å. The spin state is still a singlet, but in this case, there is no spin anywhere. The barrier from the NO_2_H reactant is quite small, with only +5.1 kcal/mol. However, since the reactant is at +14.9 kcal/mol, the barrier from the resting state with NO_2_^−^ is 14.9 + 5.1 = 20.0 kcal/mol. That is rather high, but barrier heights are generally overestimated by a few kcal/mol with the present approach. The mechanism is the same as in the previous paper [14]. No TS was reported there, but from the energies of the intermediates, the barrier is probably similar to the one obtained here.

The product of the N—O cleavage has NO bound to Fe(II) by a short bond with a distance of 1.65 Å. The water that is formed is bound by hydrogen bonds to the cofactor with a binding energy of −6.8 kcal/mol. Again, as for the NO_2_H structure, there are some spins appearing even though it is a singlet. The spin on iron is −0.24, indicating Fe(II), and NO has a spin of +0.20, probably not important for the energy. His277 is still unprotonated. The exergonicity starting from the NO_2_H structure is −10.5 kcal/mol. This means that, so far, the reaction from the starting NO_2_^−^ structure is endergonic by +4.4 kcal/mol.

After the above scenario, the first reduction takes place. Adding an (H^+^, e^−^) couple is exergonic by −16.1 kcal/mol. The proton ends up on His277 and the electron on NO. That is the first major difference compared to the previous study, in which a structure with NHO was suggested [14]. In the present study, the energy for an NHO structure is much higher in energy than the HisH^+^ structure at this stage. In fact, a geometry optimization for an NHO structure led automatically to a structure with HisH^+^. The water molecule formed after the first N—O cleavage is no longer bound to the cofactor. The state is now a doublet with most spin located on NO with 0.91. The iron is still Fe(II). The structure is shown in Figure S2. It was tested whether the reduction was proton‐coupled or not. It turned out that proton coupling was only slightly preferred by −1.3 kcal/mol.

**FIGURE 3:**
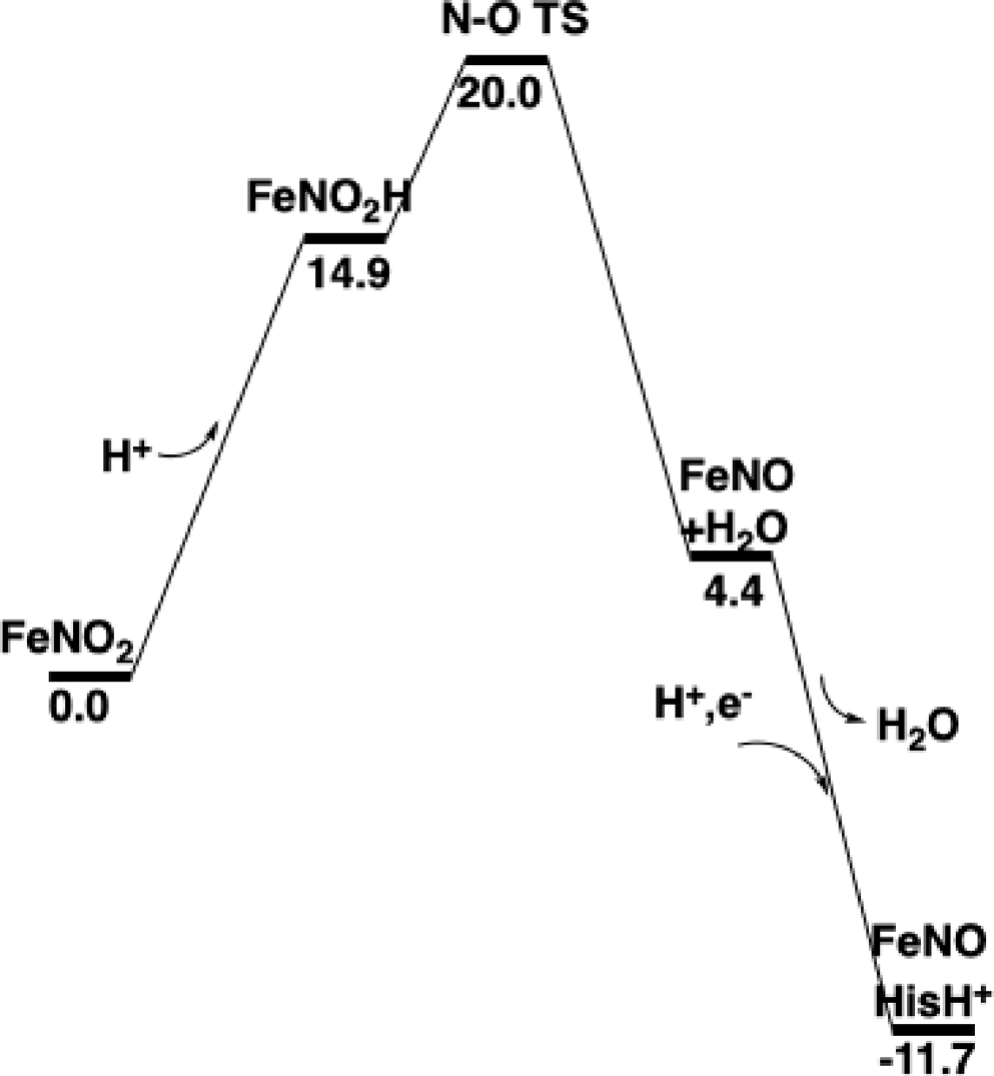
Energy diagram for the first half of nitrite reduction by nitrite reductase.

The energetics up to this point are shown in Figure 3.

The next reductions are critical for the mechanism. The first reduction needs to protonate NO to form NHO. It was tested whether a reduction without adding a proton was possible, but it was found to be endergonic by +14 kcal/mol. Therefore, the reduction is proton‐coupled also in this case. There is no other place to put the proton than on NO. At this stage, all amino acids in the active site, His277, Tyr218, and Arg114, are already protonated. In spite of many attempts, the best protonation of NO was found to be endergonic by +6.5 kcal/mol. Forming NH_2_O in this reduction step, by using the proton on HisH^+^ is even more endergonic. Ending a reduction step at an endergonic stage has, so far, not been found in any other enzyme mechanism studied. It means that two electrons need to be added to the active site quickly after each other, which puts high demands on the structure of the active site. The two reductions need to be in thermal equilibrium, implying that the electron transfer has to be very fast. That is probably the reason the active site is surrounded by four bis‐histidine heme groups, which act as electron donors.

**FIGURE 4:**
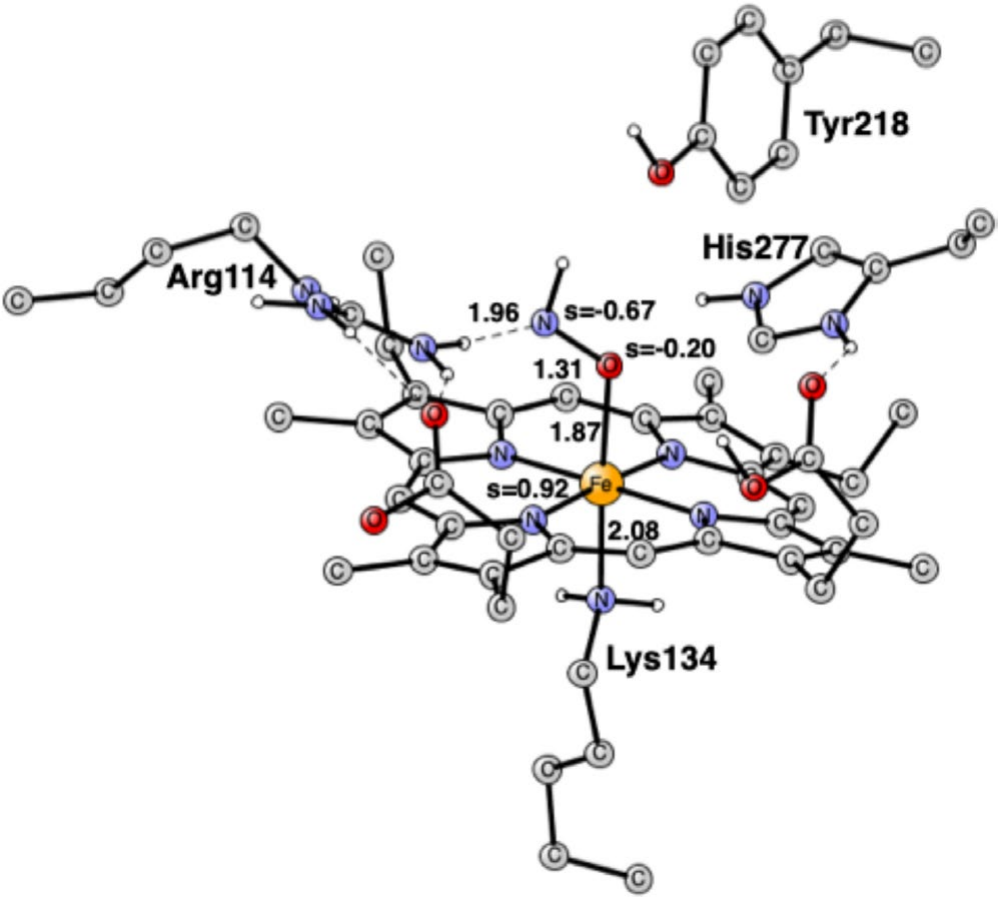
Optimized structure for the FeONH intermediate. Distances are in Å. Some spins are also given. The heme iron is Fe(III).

The optimized FeONH structure is shown in Figure 4. ONH has nearly one spin on the nitrogen with −0.67 and on the oxygen −0.20, implying an anionic ONH^−^ state of the substrate. The additional electron, apart from the one obtained from the electron donor, comes from the iron, which changes its oxidation state from Fe(II) to Fe(III).

In the next reduction, ONH_2_ is formed; see Figure S3. The proton and electron from the incoming (H^+^, e^−^) go to the substrate, which binds with its oxygen end to Fe(III). The reduction is quite exergonic at −17.9 kcal/mol. Since the transfer of just the electron is very endergonic, the reduction is strongly proton‐coupled. The formation of hydroxylamine is completed in the next reduction. There is no outside proton entering the cofactor in this step. The electron goes to iron, which becomes Fe(II), and a proton moves from His277 to the substrate. The product is a singlet, and hydroxylamine binds with its oxygen end to Fe(II) with a bond distance of 2.34 Å; see Figure S4. The formation is slightly endergonic at +3.9 kcal/mol, but this is not the end of this reduction step; see below. The transfer of just the electron without taking the proton from His277 to the substrate is very endergonic, so the reduction is also, in this case, strongly proton‐coupled.

**FIGURE 5:**
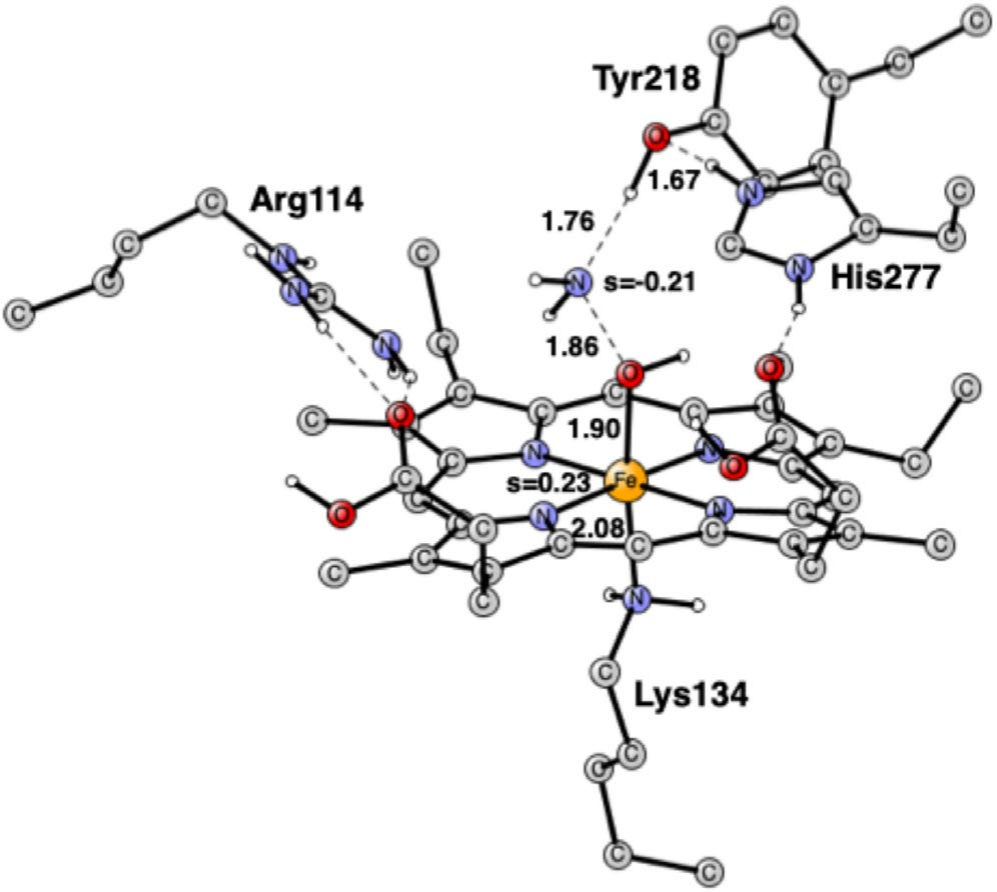
Optimized TS for the cleavage of the second N—O bond. Distances are in Å. The imaginary frequency is 593 cm^−1^.

After the formation of the hydroxylamine, the second N—O bond can be cleaved, which occurs at the singlet surface. The TS is shown in Figure 5. The N—O distance is 1.86 Å, very similar to the corresponding N—O distance when the first N—O bond is cleaved. Some spins have started to develop since iron needs to be Fe(III) when it binds the negative OH^−^. The spin on iron is +0.23, and the spin on the NH_2_ group is −0.21. The Fe—O bond is shortened to 1.90 from 2.34 Å in hydroxylamine. The H‐bond from NH_2_ to the oxygen of Tyr218 is rather short at 1.76 Å. The effect of the N—O elongation is felt all the way to His277, as seen on the H‐bond between Tyr218 and His277. The computed barrier height from the hydroxylamine is rather low at +8.8 kcal/mol. However, since the formation of the hydroxylamine is endergonic by +3.9 kcal/mol, the actual barrier height from the resting state is +12.7 kcal/mol, which is still rather low.

**FIGURE 6:**
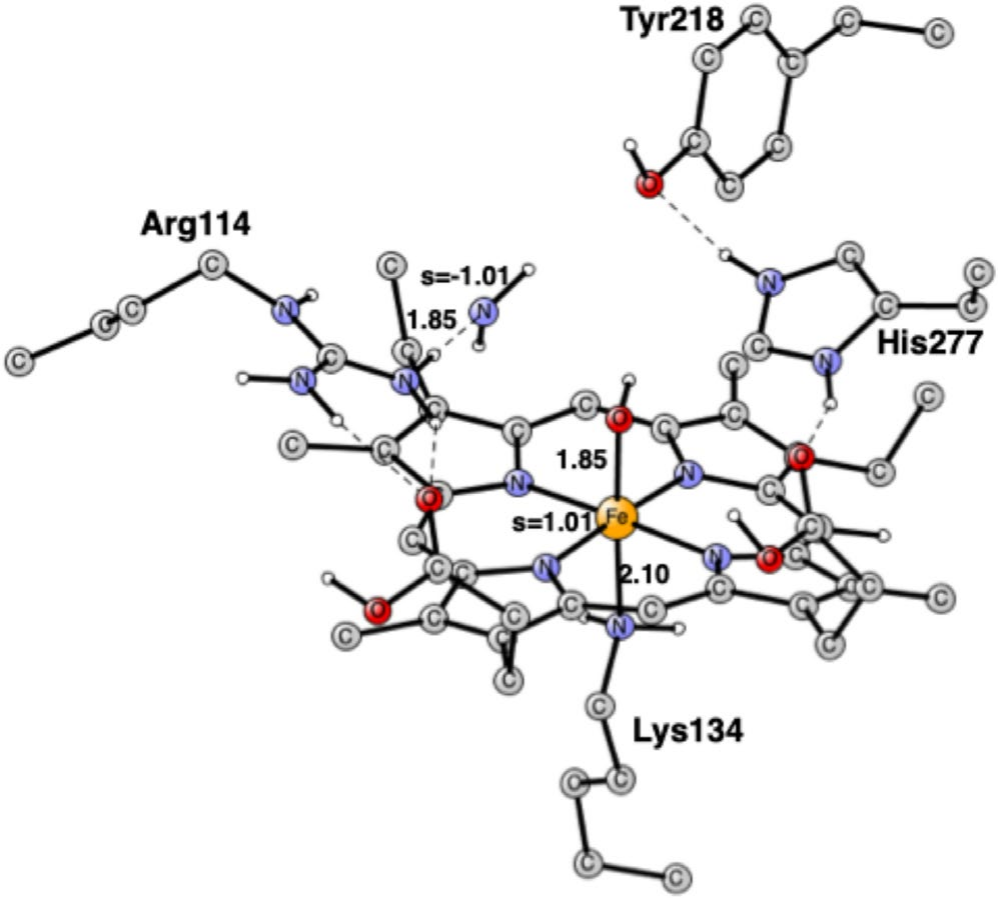
The NH_2_ product after the cleavage of the second N—O bond. There is an NH_2_ radical, and the heme iron is Fe(III).

The product after the cleavage of the second N—O bond is shown in Figure 6. The iron is now a fully formed Fe(III) with a spin of 1.01. Since the total spin is a singlet, the spin on the NH_2_ radical is −1.01. There is a rather short H‐bond between Arg114 and NH_2_. It is interesting that the structure with the NH_2_ radical is actually lower by −4.0 kcal/mol than that of the hydroxylamine.

**FIGURE 7:**
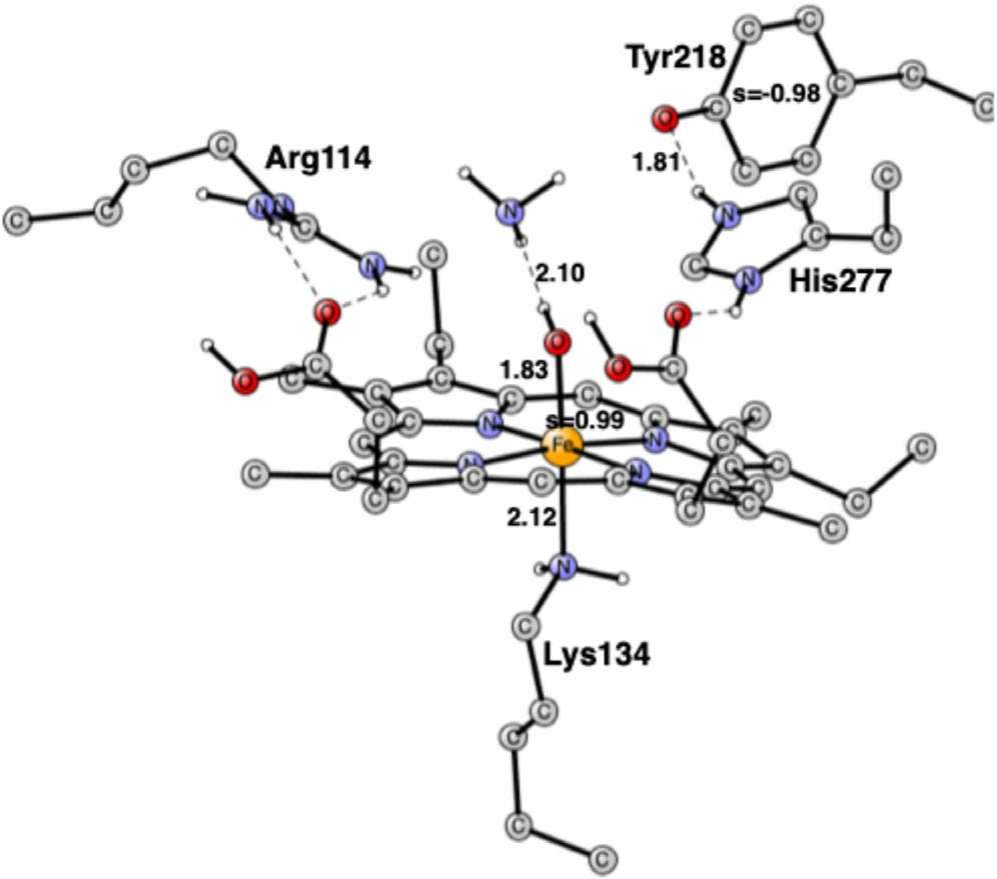
Optimized structure for the NH_3_ product intermediate after the cleavage of the second N—O bond. Distances are in Å. There is a tyrosyl radical, and the heme iron is Fe(III).

In the next step, the final NH_3_ product is formed, see Figure 7. It is only weakly bound and released in a very exergonic step by −24.0 kcal/mol. Since the NH_2_ spin disappears when NH_3_ is formed, another negative spin needs to be formed. There is only one possibility, and that is to form a radical on Tyr218. The formation of the Tyr218 radical has been suggested based on experiments, where it should be formed at a late stage of the mechanism [10], which is exactly what is found here. In the previous study, no tyrosyl radical was found anywhere in the mechanism [14]. The ammonia is not bound and leaves.

Two reductions remain to protonate the hydroxide and the tyrosyl radical. The first protonation found was that of the tyrosyl. The spin on iron, which is Fe(III), remains at 1.0. The step is strongly exergonic at −28.3 kcal/mol, and is only weakly proton coupled. In the final reduction, the OH is protonated and Fe(II)—H_2_O is formed. The spin state is then a singlet without any spin. This reduction step is exergonic at −2.5 kcal/mol.

**FIGURE 8:**
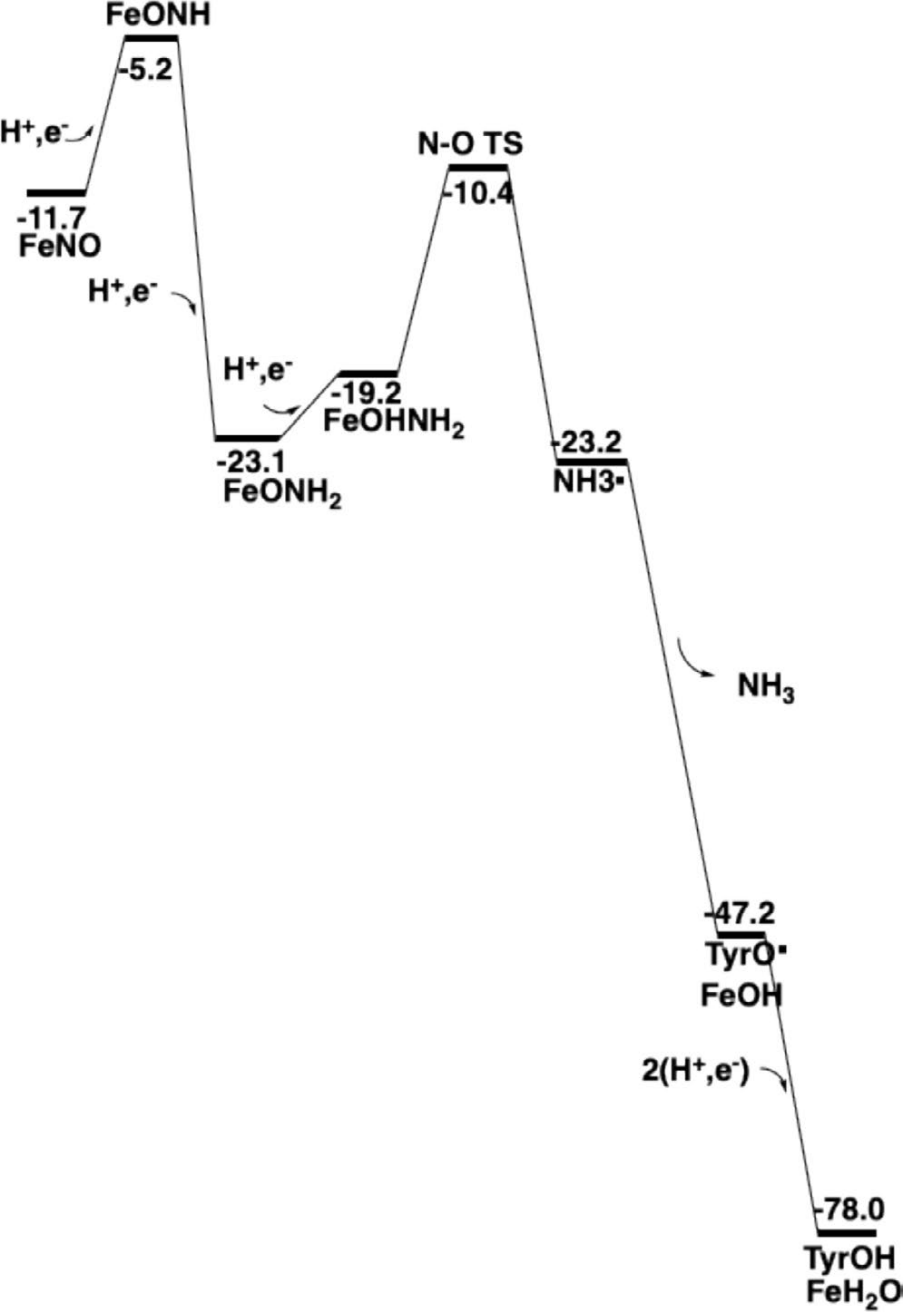
Energy diagram for the second half of the nitrite reduction by nitrite reductase.

The energetics for the second half of the nitrite reduction mechanism is shown in Figure 8. As described above, the first half‐reaction is exergonic by −11.7 kcal/mol, while the second half is much more exergonic at −66.3 kcal/mol. About half of the exergonicity comes from the two final reductions to form water and the neutral tyrosine. The formation and release of NH_3_ is also a very exergonic step. The most demanding reduction is the one for the formation of NHO, which is actually endergonic. The energy goes down only in the next reduction, when ONH_2_ is formed.

A different mechanism was finally tested, which is used by another nitrite reductase that has been studied previously [24]. In that enzyme (CuNiR), a cofactor with one copper is used instead. It produces nitric oxide and water after only one reduction. One of many differences to the mechanism studied here is that nitrite binds with an oxygen to the metal, whereas in the present enzyme (CcNiR) it binds with the nitrogen. In CcNiR, binding to nitrogen is preferred by −6.6 kcal/mol. It was still tested whether NO could be released from the Fe—ONO bound structure in CcNiR. An approximate barrier of about 20 kcal/mol was obtained for breaking the O—N bond using the proton on His277, starting from the Fe—ONO structure. However, since the Fe—ONO structure is higher by +6.6 kcal/mol than the resting state, which is the Fe—N bound ONO, the total barrier is too high to be reached. The limit for an allowed reaction is +20 kcal/mol. Therefore, the mechanism used by CuNiR, leading to a release of NO, cannot be used by CcNiR.

## 4 Conclusions

The reduction of nitrite by cytochrome c nitrite reductase (CcNiR) has been studied using a quantum chemical modeling. Computational methods have been used that have given excellent agreement with experiments for a number of redox enzymes, such as Photosystem II, Nitrogenase, and several others. The reduction of nitrite needs six reductions and seven protons to yield the final products, ammonia, and water. For each reduction, it was investigated whether the reductions are proton‐coupled or not. It was found that all of the six reductions are proton‐coupled, except possibly for the case where the first NO structure is reduced and in the step where the tyrosyl is reduced. The only step where just a proton is added is the first one when nitrite is protonated, which is a quite endergonic step by +14.9 kcal/mol. There is no reduction in that step.

As discussed in the text, there have been a few earlier computational studies of the mechanism for CcNiR [11, 12, 13, 14]. For the cleavage of the first N—O bond, the present mechanism is the same as in the previous one [14]. There is no reduction in that step. For the cleavage of the second N—O bond, there are major differences between the two studies, in particular, for the critical protonation of NO. In the previous study, NO was protonated in the first reduction step of the mechanism. In contrast, in the present study, a protonation of His277 was strongly preferred compared to the protonation of NO at that stage. The NO protonation was here instead found to occur in the second reduction, but it was found to be endergonic. That endergonicity puts strong demands on the structure of the cofactor. Most of all, in the next step, which needs to make the process exergonic, the reduction has to be quite fast. The fact that the active site is surrounded by electron‐donating bis‐histidine heme groups is most probably significant for enabling a fast reduction after the first one.

There are other important differences from the previous study. A quite surprising result in the previous study was that Arg114 was found to act as a proton donor, which is not supported by the present study. Furthermore, in the present study, Tyr218 was found to be redox active, which was not found in the previous studies. A contributing factor for these differences is that in the previous study, the substrate was assumed to be coordinated with its nitrogen to iron in all structures after the first reduction. In the present study, the only case where the substrate is coordinated by nitrogen is for NO. For NHO, NH_2_O, and NH_2_OH, coordination by oxygen was found to be preferred.

In both a previous study, where the oxidization of NH_2_OH was studied [6], and the present one, where NO is reduced, the step where NHO is involved was found to be the most critical part of the mechanism. For the step where NH_2_O is oxidized, both protons leave to form NO, which means that HNO as an intermediate is therefore avoided. In the present reduction of NO, forming HNO is found to be endergonic, requiring a fast exergonic reduction directly following its formation.

## Supporting information


**Data S1** Supporting Information.

## Data Availability

The data that support the findings of this study are available on request from the corresponding author. The data are not publicly available due to privacy or ethical restrictions.
