# Supplementary material for: The Mechanism of Nitrite Reductase
Source: J Comput Chem. 2025 Mar 24;46(8):e70088. doi: 10.1002/jcc.70088 (PMC11932435; doi:10.1002/jcc.70088)
Supplement: Supplementary file 1 — Data S1 Supporting Information. [file JCC-46-0-s001.docx]

Supporting information:

The mechanism of nitrite reductase

Per E. M. Siegbahn^*^

Department of Organic Chemistry, Arrhenius Laboratory, Stockholm University, SE-106 91, Stockholm, Sweden. Email:per.siegbahn@su.se

**Some structures discussed in the text.**

**Figure S1.** Optimized structure for the NO_2_H intermediate. Distances are in Å. Some spins are also given. The heme iron is Fe(II).

**Figure S2.** Optimized structure for the NO intermediate. Distances are in Å. Some spins are also given. The state is a doublet and the heme iron is Fe(II).

**Figure S3.** Optimized structure for the ONH_2_ intermediate. Distances are in Å. Some spins are also given. The heme iron is Fe(III).

**Figure S4.** Optimized structure for the NH_2_OH intermediate. Distances are in Å. There are no spins. The heme iron is Fe(II).

**xyz- coordinates for the structures discussed in the text.**

The structure in **Figure 1.** # means fixed atom

Energies: E= -3472.669850, solv = -0.067487, disp = -192.06 Z_0_ = 847.35

Eps=20: solv=-0.092985

Fe1 27.3331895080 56.3319625778 16.9343786723

C2 25.2636292696 53.8726747950 18.0884857954

C3 28.9486051490 54.0826963084 14.9502504429

C4 29.1145204525 58.8822614956 15.5734706127

C5 26.1949433864 58.4157309350 19.4013049472

C6 26.2657264011 53.5090283881 17.1895063438

C7 26.5209946654 52.1406130323 16.7767963318

C8 27.5620614147 52.1818347925 15.8833955024

C9 27.9516148721 53.5837694221 15.7903025618

C10 28.1569789750# 51.0510100323# 15.0929930226#

C11 25.7048189070 50.9679127289 17.2280142973

C12 24.3856225092 50.8667741322 16.4174011127

C13 24.7229790249# 50.6400110229# 14.9749950395#

O14 25.1382198467 49.3780642044 14.7631297941

O15 24.6497531060 51.4513834062 14.0601891363

C16 29.2893247232 55.4269114804 14.7985608886

C17 30.1472538431 55.9465408054 13.7445827136

C18 30.1776989715 57.3072052039 13.9011508073

C19 29.3379315549 57.6057606376 15.0563655330

C20 30.7959749053# 55.1300159934# 12.6669929876#

C21 30.8488954530 58.3081772867 13.0053796777

C22 30.0089717770# 58.6030162759# 11.7499932009#

C23 28.3592884809 59.1916219885 16.7059704885

C24 28.3724639809 60.4772767588 17.3945620568

C25 27.6611283879 60.2982577714 18.5543850093

C26 27.1236172206 58.9430684635 18.5010657448

C27 29.0409716960# 61.7380139043# 16.9269939830#

C28 27.5421732423 61.2609828097 19.7061111340

C29 26.9359740228# 62.6240159101# 19.3539939915#

C30 25.5674667581 57.1700924381 19.3054289312

C31 24.5251633168 56.6890343165 20.2014383084

C32 24.2092659253 55.4173101468 19.7832179328

C33 25.0925338328 55.1307349812 18.6613749414

C34 23.9419770310# 57.4440119678# 21.3619978922#

C35 23.2289731044 54.4634495778 20.4172528461

C36 21.7344390525 54.6697966430 20.0427686946

C37 21.3749769563# 54.3240090457# 18.6159949932#

O38 21.5868522678 53.0155761902 18.3381807316

O39 20.9336737226 55.0919222068 17.7809473015

N40 27.1531543129 54.3590649813 16.5905434941

N41 28.8156995203 56.4497152350 15.5758052346

N42 27.5882082245 58.2878236998 17.3870476339

N43 25.8702493209 56.2196963069 18.3634249664

C44 31.9819769964# 55.8750149985# 22.3859920008#

C45 32.2565121824 54.9339297852 21.2057602233

C46 31.2896826958 55.0959697147 20.0195031755

C47 29.8299830908 54.7399613213 20.3411024553

C48 28.8944938502 54.7116549575 19.1242866989

N49 28.7306562653 56.0149292779 18.4411349542

C50 30.4419729789# 51.7440150556# 8.3349930686#

C51 29.6738288728 52.8273419698 9.1120439801

C52 28.1498138547 52.6694290616 8.9992265939

C53 27.3060046277 53.8134022748 9.5937162658

N54 27.2923640383 53.9244373614 11.0660851810

C55 26.3987050530 53.3048260191 11.8628124866

N56 25.7026309123 52.2445738229 11.3900629677

N57 26.1707609978 53.7192580618 13.1001208901

C58 24.1709713348# 65.7240069997# 15.0359967810#

C59 25.2153530796 64.8270803731 15.7285107559

C60 25.4743262157 63.4898469271 15.0540777079

C61 24.5847222471 62.4131282803 15.1919441765

C62 26.6283776459 63.2663192230 14.2862963348

C63 24.8452639990 61.1627491668 14.6254096725

C64 26.9097204197 62.0223631668 13.7187801630

C65 26.0320086908 60.9459453011 13.9057933249

O66 26.3566441491 59.7412223511 13.3684981550

C67 19.2469753016# 58.8630067938# 17.5249980392#

C68 20.3048708905 58.4104763563 18.5456005406

C69 21.6994773180 58.6464368880 18.0612651674

N70 22.3476559691 59.8759008083 18.1502711568

C71 22.5707052374 57.8314776594 17.3906976978

C72 23.5547344397 59.7975408517 17.5621255325

N73 23.6977948972 58.5661772830 17.0963624205

H74 25.1281198433 51.7415499620 12.0612571433

H75 26.0669211818 51.7242093727 10.6061974241

H76 26.4939004900 54.6128339445 13.4923335367

H77 25.5951167958 53.1433027901 13.7093578183

H78 27.7761075849 54.7085607196 11.4859812730

H79 29.9721344685 52.8081460986 10.1687208407

H80 29.9574494001 53.8176521706 8.7289020042

H81 27.8755822062 52.6080101018 7.9369600093

H82 27.8522979826 51.7049448352 9.4387360227

H83 27.6767308109 54.7722135329 9.2196149542

H84 26.2671191475 53.7253928855 9.2554281950

H85 31.5228534923 51.8817927730 8.4378667522

H86 30.1991344971 51.7792748910 7.2668330914

H87 30.1969715231 50.7401820055 8.7022041741

H88 19.3276430175 59.9328343986 17.3016565538

H89 18.2408008540 58.6773116167 17.9139630043

H90 19.3573354924 58.3104302289 16.5872846350

H91 20.1508194894 58.9196683782 19.5060748679

H92 20.1971197181 57.3373138723 18.7240695124

H93 22.4644364666 56.7945971604 17.1181273597

H94 24.2769211012 60.5934233318 17.4677216337

H95 24.5383964148 58.2310302903 16.5341316203

H96 25.8988685768 59.0202139589 13.8573578612

H97 24.0372460686 66.6606956530 15.5886125366

H98 23.1971298154 65.2246648837 14.9730825836

H99 24.4814170098 65.9715939717 14.0150455215

H100 26.1625215317 65.3763416686 15.7991306932

H101 24.8939832754 64.6500758113 16.7647312868

H102 23.6579541491 62.5589908660 15.7469869450

H103 27.3360003801 64.0803758760 14.1430301225

H104 27.8176754976 61.8632567793 13.1451101574

H105 24.1312279676 60.3499788373 14.7252121448

H106 32.7383984687 55.7513071512 23.1685260820

H107 31.0043979484 55.6841197143 22.8426103345

H108 32.0025264821 56.9245664349 22.0663870177

H109 33.2801604548 55.1015573265 20.8460562611

H110 32.2240864603 53.8917209943 21.5540518644

H111 31.3503956544 56.1315568369 19.6525954358

H112 31.6343838306 54.4568854597 19.1930560600

H113 29.7984348293 53.7452859872 20.8094411922

H114 29.4244383913 55.4384049143 21.0871153174

H115 29.2566342921 53.9971155843 18.3783891555

H116 27.8970534327 54.3819760790 19.4274226063

H117 28.5045159545 56.7309675515 19.1327655875

H118 29.4436819174 62.3125537079 17.7681060084

H119 28.3400400165 62.3791204896 16.3802982427

H120 29.8778147447 61.5281689307 16.2525669618

H121 26.9609530585 60.8078493173 20.5173589976

H122 28.5444916806 61.4248484447 20.1293974580

H123 26.8679924137 63.2595236510 20.2447991614

H124 25.9238065156 62.5133047810 18.9443001804

H125 27.5285048869 63.1583700325 18.6086366692

H126 31.0357650528 59.2422432766 13.5470657235

H127 31.8371175089 57.9362709239 12.7052984278

H128 29.0188804142 58.9828961184 12.0257400254

H129 29.8669587790 57.6939943914 11.1540882829

H130 30.4998086585 59.3475408573 11.1122717541

H131 29.6208212691 59.7054159772 15.0833313151

H132 25.9001176017 59.0468805927 20.2331907943

H133 29.4631415164 53.3679088277 14.3149289748

H134 29.2476405794 51.0060137685 15.2006738741

H135 27.9506003624 51.1619303203 14.0190412752

H136 27.7568222004 50.0835571746 15.4095728632

H137 26.2607276357 50.0314735931 17.1191520139

H138 25.4477484704 51.0547144720 18.2888397194

H139 23.7793954671 50.0268354157 16.7736603577

H140 23.8141483317 51.7928182638 16.5027951997

H141 24.6149111661 53.0816550464 18.4469326207

H142 31.4365017865 55.7483549997 12.0317194595

H143 30.0601339116 54.6415175852 12.0125107768

H144 31.4216833874 54.3277603124 13.0800405111

H145 24.7214040350 57.9311839444 21.9591859165

H146 23.3864406248 56.7790727814 22.0307391076

H147 23.2487132496 58.2317507888 21.0354411284

H148 23.5029474437 53.4267517914 20.1993841404

H149 23.2878917570 54.5620568152 21.5081304594

H150 21.1251305394 54.0308714588 20.6946154217

H151 21.4391947506 55.7072514345 20.2137686445

H152 25.3688633689 49.2864580133 13.8198940880

H153 21.3024830130 52.8849966166 17.4130992351

N154 26.1686509842 56.8138279287 15.3351105257

O155 26.2745243051 56.2508277553 14.2351599316

O156 25.4670678725 57.8825595470 15.3106406077

H157 29.6118783485 56.3024339760 18.0155479643

H158 21.9736245324 60.7074591753 18.5881110961

The NO_2_H structure

Energies: E= -3473.020159, solv = -0.142671, disp = -186.86 Z_0_ = 855.58

Eps=20: solv=-0.186579

Fe1 27.3315976251 56.2950115916 16.9647186881

C2 25.3425729506 53.8094256521 18.1998656984

C3 28.9415957459 54.0696575982 14.9661388171

C4 29.0786036161 58.8824086530 15.5412054200

C5 26.1600938134 58.4125689184 19.3717755827

C6 26.3445551289 53.4523968898 17.3039626808

C7 26.5701892437 52.0997144313 16.8384949880

C8 27.5927078671 52.1598726582 15.9259050937

C9 27.9953961709 53.5564384260 15.8597520490

C10 28.1569780127# 51.0510114506# 15.0929936656#

C11 25.7322379556 50.9233887437 17.2391825538

C12 24.4087901621 50.8662109134 16.4247752175

C13 24.7229798650# 50.6400113385# 14.9749945345#

O14 24.9619438555 49.3466091003 14.7210509656

O15 24.7884324928 51.4978613418 14.0983820578

C16 29.2639037420 55.4178929960 14.7936390779

C17 30.1236738207 55.9370870737 13.7404899751

C18 30.1496541652 57.2988038845 13.8963580221

C19 29.3015860733 57.6016832391 15.0403139565

C20 30.7959741800# 55.1300165583# 12.6669939701#

C21 30.8365351514 58.2970986335 13.0102369046

C22 30.0089716754# 58.6030164801# 11.7499928529#

C23 28.3157827020 59.1952066714 16.6668312984

C24 28.3380522025 60.4768556534 17.3643874547

C25 27.6199100357 60.2909991485 18.5223067659

C26 27.0730880194 58.9419324486 18.4603527066

C27 29.0409715270# 61.7380136646# 16.9269939873#

C28 27.5111403299 61.2415499744 19.6860454621

C29 26.9359740325# 62.6240158592# 19.3539940149#

C30 25.5719501650 57.1485458098 19.3142192023

C31 24.5257785795 56.6757921027 20.2107676133

C32 24.2276178936 55.3925844172 19.8163653846

C33 25.1465091261 55.0782779426 18.7337721115

C34 23.9419773486# 57.4440116533# 21.3619973404#

C35 23.2113844633 54.4621439271 20.4282899995

C36 21.7248025697 54.7222202459 20.0299210527

C37 21.3749770393# 54.3240098905# 18.6159945294#

O38 21.2205827433 52.9993848466 18.4746371729

O39 21.2519027053 55.0872772236 17.6644745273

N40 27.2363312141 54.3091528338 16.7156334664

N41 28.7786421397 56.4443750222 15.5645560578

N42 27.5306159303 58.2882527937 17.3376134847

N43 25.9188199659 56.1666505908 18.4208467637

C44 31.9819766317# 55.8750149904# 22.3859915871#

C45 32.3048642828 54.9999276847 21.1699934090

C46 31.3368735478 55.1856507598 19.9899316233

C47 29.8782957005 54.8536028823 20.3374609382

C48 28.9316622740 54.7835517173 19.1372842120

N49 28.7692489659 56.0698186167 18.4090540168

C50 30.4419719415# 51.7440156027# 8.3349940021#

C51 29.5158758938 52.8099459255 8.9350781835

C52 28.0302463826 52.4360418291 8.8270464761

C53 27.0645455332 53.5468101598 9.2629546515

N54 27.0853821915 53.8690136216 10.7040832076

C55 26.3506960350 53.2807199842 11.6570481672

N56 25.4944879307 52.2899135252 11.3760647540

N57 26.4221799554 53.7133927429 12.9299402433

C58 24.1709721793# 65.7240048968# 15.0359965798#

C59 25.2876494248 65.2958282670 14.0689663371

C60 25.3570318441 63.7911593546 13.9327822732

C61 25.8029261220 63.0101284609 15.0096637961

C62 24.9227993746 63.1313261450 12.7753290310

C63 25.8009051179 61.6176634124 14.9488460577

C64 24.9251257259 61.7367031163 12.6883713683

C65 25.3607117084 60.9931150170 13.7813322107

O66 25.2845527570 59.5986334190 13.7017577067

C67 19.2469765669# 58.8630056145# 17.5249979361#

C68 20.6164391637 59.3817875665 17.9862874590

C69 21.6727408569 59.0177799311 16.9986311813

N70 21.9911963237 57.6943095431 16.7170884928

C71 22.4129931942 59.7730445101 16.1310666005

C72 22.8792500989 57.6506540419 15.7235633310

N73 23.1466326138 58.9009438094 15.3475120094

H74 25.0344180080 51.8118154567 12.1493025165

H75 25.3250878360 51.9821881107 10.4312544331

H76 27.2569760340 54.1797207169 13.2775514359

H77 25.8951344356 53.2027845997 13.6367867268

H78 27.6691843119 54.6400830674 11.0000975277

H79 29.7811039715 52.9757905332 9.9891223530

H80 29.6811136137 53.7663915297 8.4187179901

H81 27.7908073013 52.2036832076 7.7807793631

H82 27.8273190646 51.5199248154 9.3990287026

H83 27.3057113142 54.4799953491 8.7452574496

H84 26.0319873908 53.3108773684 8.9805865409

H85 31.4914542179 52.0389791709 8.4301407709

H86 30.2326193237 51.5959035712 7.2698336388

H87 30.3190334240 50.7784955431 8.8391446319

H88 18.9745802502 59.2861588398 16.5532062246

H89 18.4712763335 59.1417930224 18.2446715954

H90 19.2405389112 57.7710399328 17.4364989744

H91 20.5994506349 60.4706250559 18.0947125493

H92 20.8662798093 58.9719629891 18.9736646660

H93 21.6462572720 56.8496111951 17.1977956492

H94 23.3321809780 56.7558581473 15.3283690720

H95 24.1377017868 66.8141001836 15.1361267489

H96 24.3270209130 65.3009157563 16.0347416712

H97 23.1919020375 65.3876034909 14.6772390723

H98 25.1220791349 65.7511558101 13.0861030173

H99 26.2492854913 65.6809911820 14.4319663749

H100 26.1427676829 63.4986523049 15.9197405716

H101 24.5782804527 63.7142458793 11.9253283253

H102 24.5949789547 61.2289944804 11.7875316153

H103 26.1379248800 61.0264007828 15.7963588771

H104 32.7450987398 55.7588649708 23.1626228757

H105 31.0180917200 55.6140521356 22.8367276504

H106 31.9497287178 56.9377351267 22.1129082515

H107 33.3234892534 55.2178146805 20.8252620307

H108 32.3041604266 53.9424821828 21.4689585157

H109 31.4105384359 56.2235435713 19.6302794359

H110 31.6615579045 54.5445774855 19.1568541588

H111 29.8411519280 53.8751752135 20.8372174878

H112 29.4855410998 55.5777517724 21.0647387643

H113 29.2843258810 54.0444875787 18.4126188882

H114 27.9352338079 54.4742001180 19.4605324274

H115 28.5673890366 56.8104881944 19.0828487682

H116 29.4359414844 62.2871624929 17.7863856236

H117 28.3677011676 62.4063507677 16.3786942927

H118 29.8874045015 61.5224120335 16.2671944608

H119 26.9156562510 60.7902005781 20.4866092908

H120 28.5156333910 61.3722217711 20.1143777617

H121 26.8760728290 63.2379295508 20.2587015639

H122 25.9242597528 62.5400674668 18.9398466871

H123 27.5501641322 63.1647749127 18.6306200420

H124 31.0331311305 59.2268380107 13.5548267834

H125 31.8208359793 57.9142390836 12.7147508069

H126 29.0282355192 59.0139088140 12.0151279216

H127 29.8469239446 57.6950380517 11.1581598657

H128 30.5194800836 59.3320279146 11.1117755400

H129 29.6065757293 59.6982050340 15.0615344073

H130 25.8472610867 59.0570200194 20.1852116515

H131 29.4620936055 53.3541883452 14.3361343196

H132 29.2427047033 50.9580436505 15.2168359724

H133 27.9714384453 51.2126143717 14.0225521476

H134 27.7188813132 50.0854297334 15.3602322013

H135 26.2766158080 49.9841680103 17.1011805153

H136 25.4729055639 50.9718693258 18.3016511497

H137 23.7844824889 50.0420059315 16.7831146969

H138 23.8645025314 51.8082298840 16.5200638383

H139 24.7001616428 53.0160462318 18.5630792646

H140 31.4941669229 55.7427915786 12.0914408628

H141 30.0756821866 54.7020407481 11.9537638046

H142 31.3644244077 54.2887735544 13.0816983284

H143 24.7274854466 57.8536417035 22.0078618939

H144 23.3084113172 56.8071449668 21.9857244065

H145 23.3295444858 58.2922757266 21.0279885522

H146 23.4537958337 53.4171152969 20.2123144602

H147 23.2523273814 54.5533932357 21.5196470140

H148 21.0835975835 54.1481669109 20.7069490248

H149 21.4868101943 55.7822010550 20.1459188609

H150 25.1924737109 49.2423928016 13.7785448761

H151 20.9699385009 52.8308485933 17.5453680715

H152 29.6474123330 56.3330456950 17.9603387218

H153 22.4692221538 60.8415916218 15.9980206467

H154 23.8584334186 59.1625429693 14.6387162643

H155 25.9908223872 59.1941107150 14.2422726476

N156 26.0626625656 56.3811820109 15.5937010561

O157 25.1074875020 55.6519365165 15.3850165662

O158 26.2976493281 57.2597482874 14.4997302545

H159 25.6513028725 56.9599615326 13.8236405865

The TS for the cleavage of the first N-O bond in **Figure 2.**

Energies: E= -3473.000054, solv = -0.145257, disp = -189.93 Z_0_ = 852.80

Fe1 27.3467620000 56.3148360000 16.9999720000

C2 25.3574910000 53.8358520000 18.1961540000

C3 28.9270420000 54.0747340000 14.9381830000

C4 29.0894030000 58.8777670000 15.5696490000

C5 26.2627430000 58.3963080000 19.4714840000

C6 26.3404680000 53.4729030000 17.2828250000

C7 26.5632610000 52.1177240000 16.8247680000

C8 27.5807650000 52.1709270000 15.9075720000

C9 27.9788940000 53.5674140000 15.8265840000

C10 28.1569790000 51.0510100000 15.0929930000

C11 25.7280390000 50.9438980000 17.2376610000

C12 24.4049780000 50.8778380000 16.4228100000

C13 24.7229790000 50.6400110000 14.9749950000

O14 24.9866190000 49.3483950000 14.7377280000

O15 24.7705400000 51.4876740000 14.0885730000

C16 29.2534220000 55.4196900000 14.7790070000

C17 30.1175750000 55.9425750000 13.7349890000

C18 30.1461750000 57.3032620000 13.8985180000

C19 29.2996290000 57.6020990000 15.0442440000

C20 30.7959750000 55.1300160000 12.6669930000

C21 30.8320420000 58.3046200000 13.0152050000

C22 30.0089720000 58.6030160000 11.7499930000

C23 28.3558550000 59.1832810000 16.7194070000

C24 28.3793490000 60.4700080000 17.4049610000

C25 27.6837260000 60.2910410000 18.5768580000

C26 27.1562110000 58.9330480000 18.5456670000

C27 29.0409720000 61.7380140000 16.9269940000

C28 27.5660950000 61.2712520000 19.7150190000

C29 26.9359740000 62.6240160000 19.3539940000

C30 25.6586570000 57.1422960000 19.3911480000

C31 24.5860470000 56.6697300000 20.2552220000

C32 24.2762660000 55.3995040000 19.8414870000

C33 25.1990120000 55.0933840000 18.7616840000

C34 23.9419770000 57.4440120000 21.3619980000

C35 23.2401370000 54.4748730000 20.4315420000

C36 21.7671580000 54.7501670000 20.0167060000

C37 21.3749770000 54.3240090000 18.6159950000

O38 21.5797870000 52.9992720000 18.4007660000

O39 20.9177120000 55.0520350000 17.7600670000

N40 27.2271230000 54.3276720000 16.6848840000

N41 28.7739850000 56.4425830000 15.5621270000

N42 27.5946150000 58.2769670000 17.4184560000

N43 25.9965260000 56.1748500000 18.4814040000

C44 31.9819770000 55.8750150000 22.3859920000

C45 32.3599830000 55.0499260000 21.1504260000

C46 31.4006430000 55.2139510000 19.9586100000

C47 29.9624020000 54.7641730000 20.2578960000

C48 29.0329270000 54.7034990000 19.0413190000

N49 28.8058050000 56.0248280000 18.3930610000

C50 30.4419730000 51.7440150000 8.3349930000

C51 29.3765790000 52.7518310000 8.7855200000

C52 27.9486000000 52.1961270000 8.6810450000

C53 26.8491030000 53.2096960000 9.0285040000

N54 26.8422890000 53.6523260000 10.4369790000

C55 26.2228080000 53.0510930000 11.4612880000

N56 25.4092450000 52.0015660000 11.2734910000

N57 26.4007180000 53.4936310000 12.7140280000

C58 24.1709710000 65.7240070000 15.0359970000

C59 23.3829970000 64.5600390000 14.4178970000

C60 24.1419780000 63.2479410000 14.3258070000

C61 25.0146420000 62.8308190000 15.3393280000

C62 23.9730930000 62.3938110000 13.2251940000

C63 25.6844730000 61.6094780000 15.2646910000

C64 24.6389830000 61.1704470000 13.1305820000

C65 25.4968170000 60.7847960000 14.1571110000

O66 26.1291660000 59.5422450000 14.0679350000

C67 19.2469750000 58.8630070000 17.5249980000

C68 20.0868510000 59.6475230000 18.5407100000

C69 21.5444010000 59.5641410000 18.2075590000

N70 22.4190730000 60.6336240000 18.3153980000

C71 22.3006680000 58.5469390000 17.6805930000

C72 23.6344970000 60.2560780000 17.8570660000

N73 23.5912830000 58.9913710000 17.4734200000

H74 24.9654410000 51.5920400000 12.0936470000

H75 25.0473980000 51.7766300000 10.3594370000

H76 27.1701600000 54.0980940000 12.9730620000

H77 25.8850230000 53.0540160000 13.4727660000

H78 27.3859780000 54.4742420000 10.6620680000

H79 29.5801640000 53.0580490000 9.8225390000

H80 29.4529790000 53.6606570000 8.1718080000

H81 27.7617620000 51.8674300000 7.6501700000

H82 27.8405380000 51.3046890000 9.3137810000

H83 26.9631260000 54.1181660000 8.4294790000

H84 25.8572490000 52.8159260000 8.7816100000

H85 31.4461560000 52.1707060000 8.4209260000

H86 30.2919460000 51.4531750000 7.2893330000

H87 30.4119260000 50.8322840000 8.9431470000

H88 19.4003560000 59.2449020000 16.5109570000

H89 18.1823010000 58.9463890000 17.7651640000

H90 19.5067270000 57.7990630000 17.5337640000

H91 19.7766990000 60.7002200000 18.5512880000

H92 19.9003420000 59.2659580000 19.5538420000

H93 22.0031370000 57.5373450000 17.4352790000

H94 24.4896550000 60.9095760000 17.7998410000

H95 24.4272390000 58.3019910000 16.6531740000

H96 26.9671070000 59.5749550000 14.5676610000

H97 23.5767710000 66.6427570000 15.0026670000

H98 25.1038750000 65.9054670000 14.4914660000

H99 24.4237470000 65.5429730000 16.0865640000

H100 22.4630620000 64.4040170000 15.0014200000

H101 23.0474650000 64.8420990000 13.4130250000

H102 25.1838630000 63.4660270000 16.2042350000

H103 23.3105960000 62.6944080000 12.4173330000

H104 24.5080320000 60.5252310000 12.2675490000

H105 26.3429490000 61.2929950000 16.0680790000

H106 32.7410610000 55.7711160000 23.1681430000

H107 31.0258990000 55.5579850000 22.8176810000

H108 31.9039660000 56.9425780000 22.1426670000

H109 33.3689200000 55.3310100000 20.8238080000

H110 32.4128300000 53.9864800000 21.4215350000

H111 31.4082680000 56.2686210000 19.6428790000

H112 31.7872420000 54.6319190000 19.1089250000

H113 29.9842820000 53.7562230000 20.6952890000

H114 29.5104030000 55.4132600000 21.0206620000

H115 29.4355210000 54.0327840000 18.2774530000

H116 28.0541510000 54.3192490000 19.3356400000

H117 28.5861840000 56.7129140000 19.1148690000

H118 29.4691620000 62.2992790000 17.7630990000

H119 28.3342590000 62.3983760000 16.4107920000

H120 29.8594610000 61.5346240000 16.2290260000

H121 27.0053480000 60.8239890000 20.5426610000

H122 28.5741280000 61.4527320000 20.1147660000

H123 26.8906350000 63.2686440000 20.2380690000

H124 25.9100480000 62.4993580000 18.9848920000

H125 27.5029210000 63.1563720000 18.5874200000

H126 31.0215010000 59.2367220000 13.5586510000

H127 31.8197100000 57.9258760000 12.7257480000

H128 29.0179550000 58.9948140000 12.0068610000

H129 29.8663800000 57.6963050000 11.1515850000

H130 30.5136950000 59.3437170000 11.1207210000

H131 29.6231130000 59.6947710000 15.0972160000

H132 25.9574030000 59.0330240000 20.2938680000

H133 29.4385620000 53.3619620000 14.2986060000

H134 29.2436440000 50.9721540000 15.2183330000

H135 27.9696750000 51.1929670000 14.0203690000

H136 27.7289920000 50.0861290000 15.3775360000

H137 26.2753080000 50.0055060000 17.1062720000

H138 25.4676810000 51.0003950000 18.2992340000

H139 23.7859000000 50.0530050000 16.7884410000

H140 23.8542000000 51.8165040000 16.5127660000

H141 24.6906810000 53.0560040000 18.5429670000

H142 31.5236570000 55.7312710000 12.1159700000

H143 30.0815680000 54.7333640000 11.9306750000

H144 31.3327760000 54.2699410000 13.0847610000

H145 24.6765450000 57.9991940000 21.9565020000

H146 23.4026310000 56.7843450000 22.0480290000

H147 23.2193920000 58.1747770000 20.9746840000

H148 23.4778480000 53.4295640000 20.2136030000

H149 23.2787590000 54.5633830000 21.5236420000

H150 21.1157120000 54.2030270000 20.7097460000

H151 21.5326430000 55.8131810000 20.1043200000

H152 25.2171820000 49.2340160000 13.7964730000

H153 21.2285590000 52.8160400000 17.5080810000

N154 26.1770260000 56.4604270000 15.6669400000

O155 25.9581910000 55.9717140000 14.6464750000

O156 24.8445770000 57.7434920000 15.7604370000

H157 29.6678560000 56.3463880000 17.9517460000

H158 22.1761100000 61.5568140000 18.6493310000

H159 25.2159850000 58.3993990000 15.1163980000

The NO product structure after the first N-O cleavage, with H_2_O

Energies: E= -3473.039788, solv = -0.142804, disp = -183.75 Z_0_ = 854.09

Fe1 27.3059554074 56.3455573877 16.9542965283

C2 25.3098989318 53.8509673805 18.1569610468

C3 28.9112280553 54.0808684672 14.9244050689

C4 29.0528107466 58.8951814347 15.5379053484

C5 26.2479391285 58.4134482696 19.4554087988

C6 26.2943947113 53.4895175659 17.2440137075

C7 26.5385075451 52.1347214444 16.8021205119

C8 27.5681092536 52.1833781686 15.8933411934

C9 27.9542653647 53.5791761770 15.7966053357

C10 28.1569779443# 51.0510115612# 15.0929937177#

C11 25.7243592280 50.9520817307 17.2361591594

C12 24.4014780253 50.8398553562 16.4292735840

C13 24.7229799870# 50.6400113677# 14.9749944479#

O14 24.9330054531 49.3468649422 14.6923374041

O15 24.8253325928 51.5255788327 14.1333572959

C16 29.2251001523 55.4248766351 14.7630622659

C17 30.0955637510 55.9436482309 13.7280165992

C18 30.1148234829 57.3108636422 13.8868364821

C19 29.2565731929 57.6196085196 15.0162933461

C20 30.7959741157# 55.1300166099# 12.6669940548#

C21 30.8226543790 58.3075659023 13.0178092083

C22 30.0089716669# 58.6030164902# 11.7499928413#

C23 28.3407934052 59.1944512753 16.6940728067

C24 28.3717400106 60.4710204307 17.3888990656

C25 27.6835794763 60.2902319758 18.5660336853

C26 27.1498007895 58.9393796378 18.5345225152

C27 29.0409714899# 61.7380135901# 16.9269939948#

C28 27.5719700320 61.2697974459 19.7064862202

C29 26.9359740388# 62.6240158346# 19.3539940142#

C30 25.6466586719 57.1605740816 19.3778294204

C31 24.5798298342 56.6874740358 20.2400087491

C32 24.2642346751 55.4179675823 19.8223073626

C33 25.1728452964 55.1031024002 18.7391400511

C34 23.9419773902# 57.4440116085# 21.3619973013#

C35 23.2063733278 54.5269681741 20.4194579245

C36 21.7550768123 54.8622058650 19.9736104677

C37 21.3749771251# 54.3240100973# 18.6159944493#

O38 21.5330400806 52.9825939812 18.5464625437

O39 20.9502415823 54.9597549115 17.6685668442

N40 27.1803416363 54.3466090791 16.6402337431

N41 28.7314032135 56.4564376542 15.5355623767

N42 27.5948931453 58.2807733136 17.4065900954

N43 25.9863801142 56.1816129036 18.4739268197

C44 31.9819766039# 55.8750149900# 22.3859915538#

C45 32.3221682215 55.0719659475 21.1232027953

C46 31.3514314636 55.2736051526 19.9441098699

C47 29.9133150026 54.8169356950 20.2385809455

C48 28.9900424317 54.7259713617 19.0169109787

N49 28.7717942228 56.0354434961 18.3371199649

C50 30.4419718444# 51.7440155918# 8.3349940943#

C51 29.4618238697 52.8501769549 8.7621456975

C52 28.0114404981 52.3512989137 8.8499507793

C53 26.9424272893 53.4272223426 9.1172699840

N54 26.9359604963 54.0237370585 10.4729171695

C55 26.2980085859 53.4934725118 11.5313763743

N56 25.8888975926 52.2158794737 11.5125524805

N57 26.0700239635 54.2349572230 12.6214384483

C58 24.1709722029# 65.7240047234# 15.0359965879#

C59 25.4813511000 64.9844240491 14.6708550691

C60 25.3590225664 63.4992031900 14.3490634974

C61 25.4292119475 62.5243592799 15.3586472493

C62 25.2225447020 63.0387935101 13.0285940017

C63 25.3684434304 61.1564613278 15.0685860460

C64 25.1495623516 61.6750831329 12.7238995701

C65 25.2213189427 60.7222269927 13.7443483774

O66 25.1382353462 59.3669366259 13.4404330844

C67 19.2469765909# 58.8630055347# 17.5249979432#

C68 20.7306369336 59.1483945646 17.7986969235

C69 21.5980327960 58.6943219402 16.6707130607

N70 21.5834860739 57.3913936788 16.2084393246

C71 22.4599224709 59.3570898125 15.8296305815

C72 22.4134478624 57.3184993264 15.1397476351

N73 22.9678236861 58.4921933039 14.8818573036

H74 25.4136122405 51.8224710690 12.3255659765

H75 26.1493360743 51.6053886185 10.7537133313

H76 26.2464541893 55.2502274942 12.6435931476

H77 25.6912877971 53.7809400592 13.4435484098

H78 27.1673760332 55.0066143370 10.5550954532

H79 29.7709798980 53.2666223190 9.7312876632

H80 29.5100982760 53.6769679652 8.0399620144

H81 27.7375140999 51.8833953097 7.8948336952

H82 27.9489893890 51.5501004791 9.6018045583

H83 27.0797910435 54.2611180793 8.4236807718

H84 25.9412793786 53.0239600128 8.9217149625

H85 31.4647060980 52.1290575171 8.2791551837

H86 30.1807508606 51.3471118668 7.3478848417

H87 30.4364497871 50.9092248598 9.0457585532

H88 18.9211494191 59.3475423128 16.5994150064

H89 18.6231851971 59.2384407596 18.3427860407

H90 19.0550716953 57.7884034915 17.4311249767

H91 20.8894807870 60.2220816887 17.9443794949

H92 21.0331742960 58.6609723143 18.7377334114

H93 21.1148108405 56.6047345798 16.6530614150

H94 22.5592594839 56.4096875489 14.5737367651

H95 24.3822769103 66.7746016352 15.2593699955

H96 23.6937181757 65.2767337247 15.9153651271

H97 23.4525387524 65.6881549477 14.2099088095

H98 25.9399338747 65.4909856138 13.8126782090

H99 26.1881797048 65.1024445697 15.5028887177

H100 25.5440926475 62.8379956100 16.3944679645

H101 25.1724299816 63.7602255210 12.2164572484

H102 25.0282807309 61.3434116563 11.6970801773

H103 25.4346995195 60.4230243774 15.8675933429

H104 32.7456238223 55.7248755884 23.1555171074

H105 31.0209718375 55.5747344105 22.8189772384

H106 31.9348400323 56.9501757181 22.1718442901

H107 33.3309220476 55.3418629101 20.7869936709

H108 32.3607783068 54.0016721937 21.3690190265

H109 31.3582538790 56.3361387263 19.6555243128

H110 31.7323043980 54.7133848793 19.0770330487

H111 29.9380353636 53.8146515899 20.6884366269

H112 29.4502987063 55.4709204049 20.9906685361

H113 29.3952286053 54.0383512498 18.2699786218

H114 28.0081222914 54.3538865122 19.3154186838

H115 28.5720575505 56.7452442118 19.0441555185

H116 29.4355092008 62.3057193329 17.7738917303

H117 28.3317447583 62.3757208396 16.3863147332

H118 29.8791423206 61.5368383573 16.2526571092

H119 27.0183594601 60.8199464755 20.5374572287

H120 28.5840710997 61.4473299948 20.0979519750

H121 26.8990015419 63.2623877513 20.2424433374

H122 25.9098376258 62.4939495814 18.9923112115

H123 27.4980114394 63.1588390387 18.5859396681

H124 31.0114216676 59.2377813509 13.5635123538

H125 31.8093801031 57.9200308812 12.7391918165

H126 29.0301858483 59.0304018112 11.9981716754

H127 29.8443894623 57.6906737569 11.1661386812

H128 30.5274011736 59.3213113502 11.1064961687

H129 29.5555862845 59.7173711919 15.0442690545

H130 25.9365467220 59.0579259231 20.2683410257

H131 29.4204046006 53.3721239225 14.2812799787

H132 29.2396485204 50.9677226003 15.2417610122

H133 27.9859627650 51.1957926311 14.0188214108

H134 27.7139243547 50.0928061454 15.3752025248

H135 26.2930046088 50.0244779325 17.1195417749

H136 25.4704937753 51.0211411796 18.2987049441

H137 23.8157568788 49.9899531126 16.7904584008

H138 23.8158577972 51.7564496901 16.5330738801

H139 24.6407049892 53.0731527386 18.5027870851

H140 31.4976173291 55.7460690568 12.0997951869

H141 30.0811324018 54.6964034189 11.9552873399

H142 31.3648385432 54.3004342552 13.1027606633

H143 24.6854391230 57.9636614873 21.9770171989

H144 23.3826213884 56.7778549196 22.0240000857

H145 23.2423918028 58.2058008096 20.9932515984

H146 23.4073805594 53.4717409243 20.2156407430

H147 23.2400692443 54.6288689208 21.5095374956

H148 21.0595269399 54.4110542070 20.6922964114

H149 21.5820051223 55.9401916872 19.9703892398

H150 25.1526962337 49.2539775688 13.7462506031

H151 21.1825004140 52.7031678791 17.6786277374

H152 29.6294178001 56.3369184225 17.8716544057

H153 22.7342732189 60.4014968059 15.8402716979

H154 24.3209228924 59.0008096608 13.8951742091

H155 26.1223537044 57.8712288939 12.7648396837

N156 26.0824529097 56.5671931034 15.8747787890

O157 25.2079884692 56.6014062607 15.1546541258

O158 26.6530605811 57.0454311199 12.6499047836

H159 27.5615466324 57.3013146457 12.8753154267

The NO product structure after the first N-O cleavage, without H_2_O

Energies: E= -3396.590794, solv = -0.147952, disp = -179.50 Z_0_ = 838.92

Eps=20: solv=-0.194080

Fe1 27.3368599546 56.3353975265 16.9803486626

C2 25.3140027870 53.8598661732 18.1503833047

C3 28.9120922157 54.0844684930 14.9133740887

C4 29.1047211616 58.8879376894 15.5744370563

C5 26.2688785402 58.4099709335 19.4709211037

C6 26.2954815410 53.4989595962 17.2350827663

C7 26.5352164983 52.1429366243 16.7957732703

C8 27.5625347673 52.1877245882 15.8837166299

C9 27.9515425095 53.5847687001 15.7900730316

C10 28.1569779090# 51.0510115773# 15.0929937298#

C11 25.7122437997 50.9691412781 17.2339758725

C12 24.3934170810 50.8781667086 16.4195143466

C13 24.7229801047# 50.6400113956# 14.9749943932#

O14 25.0004055921 49.3505554764 14.7451506279

O15 24.7635252618 51.4837231683 14.0835753705

C16 29.2516751971 55.4268007345 14.7678280002

C17 30.1177832629 55.9496615389 13.7326344885

C18 30.1484147845 57.3140782274 13.8976638329

C19 29.3090909355 57.6180731347 15.0432124725

C20 30.7959741034# 55.1300166094# 12.6669940643#

C21 30.8363697046 58.3130536433 13.0144450345

C22 30.0089716681# 58.6030164821# 11.7499928536#

C23 28.3859483339 59.1870655008 16.7297594733

C24 28.3950024104 60.4704689397 17.4106535720

C25 27.7045417133 60.2935959726 18.5870637098

C26 27.1826827609 58.9379097309 18.5625782603

C27 29.0409714774# 61.7380135590# 16.9269939997#

C28 27.5797743105 61.2783728816 19.7205220968

C29 26.9359740408# 62.6240158242# 19.3539940114#

C30 25.6617913811 57.1596772197 19.3815221168

C31 24.5883789782 56.6896528570 20.2408542329

C32 24.2715256620 55.4222560553 19.8191540498

C33 25.1812511466 55.1109071745 18.7352094135

C34 23.9419773865# 57.4440116067# 21.3619972943#

C35 23.2138636923 54.5281430625 20.4126332573

C36 21.7597751782 54.8612563644 19.9725453126

C37 21.3749771662# 54.3240101606# 18.6159944107#

O38 21.5048130107 52.9799806676 18.5522728230

O39 20.9705359139 54.9635969507 17.6612777398

N40 27.1785338292 54.3556453345 16.6291979928

N41 28.7854367959 56.4518096507 15.5616809216

N42 27.6475856868 58.2741825162 17.4481930482

N43 25.9966032603 56.1856599844 18.4688237043

C44 31.9819766001# 55.8750149894# 22.3859915494#

C45 32.3186465710 55.0250030359 21.1535468103

C46 31.3598784838 55.2059898106 19.9618530750

C47 29.9147192776 54.7743471931 20.2572593957

C48 28.9917857339 54.6973616232 19.0348231614

N49 28.7946005758 56.0106964739 18.3565710889

C50 30.4419717791# 51.7440155822# 8.3349941427#

C51 29.3786375812 52.7504138389 8.7939186672

C52 27.9473419553 52.2064542122 8.6703788338

C53 26.8544420775 53.2269465404 9.0169299791

N54 26.8453336206 53.6526219613 10.4316943596

C55 26.2040588725 53.0583469581 11.4440793168

N56 25.3708025570 52.0269596874 11.2494912353

N57 26.3728998096 53.5040988152 12.7029204444

C58 24.1709722142# 65.7240046965# 15.0359965849#

C59 25.6017979842 65.1980243652 14.7891976415

C60 25.6427170055 63.7078270896 14.5166184065

C61 25.4383354958 62.7868429673 15.5581306356

C62 25.8223411173 63.1924575956 13.2226019698

C63 25.3593701189 61.4167639352 15.3158390831

C64 25.7431933814 61.8239440249 12.9613063824

C65 25.4701941722 60.9260091973 14.0024647447

O66 25.3108690827 59.6016852127 13.7149753795

C67 19.2469765504# 58.8630055164# 17.5249979663#

C68 20.6706861009 59.2309744684 17.9652163664

C69 21.6899687039 58.8135586796 16.9558916452

N70 21.8373274636 57.4989909321 16.5531631371

C71 22.5832628761 59.5188926857 16.1883164836

C72 22.7865890175 57.4637146982 15.5868412581

N73 23.2674091143 58.6697105416 15.3399631037

H74 24.9243203870 51.6126350496 12.0653111260

H75 25.0251460451 51.7925544031 10.3311377373

H76 27.1980390607 54.0276858781 12.9700202846

H77 25.8758726480 53.0301393223 13.4548331630

H78 27.3749739739 54.4822746165 10.6638954521

H79 29.5758782104 53.0386136464 9.8373857885

H80 29.4651382103 53.6691039979 8.1965540543

H81 27.7673171252 51.8864977599 7.6356317479

H82 27.8256064329 51.3109531521 9.2949896594

H83 26.9842986719 54.1418908966 8.4315196527

H84 25.8595066706 52.8492795310 8.7575744358

H85 31.4477439001 52.1624027158 8.4392564222

H86 30.3004175618 51.4755222910 7.2824389211

H87 30.3996894578 50.8213697291 8.9254537077

H88 19.0034409982 59.3306712728 16.5659469814

H89 18.5127242172 59.2004446250 18.2638551977

H90 19.1286350343 57.7794148460 17.4128548827

H91 20.7532827470 60.3132675118 18.1106982445

H92 20.8858365546 58.7723991115 18.9421287161

H93 21.3549417590 56.6879807070 16.9382938187

H94 23.0712678419 56.5507601819 15.0828314737

H95 24.1846554223 66.8015674828 15.2309686191

H96 23.7113784068 65.2282920943 15.8982963276

H97 23.5323111787 65.5431090656 14.1649678565

H98 26.0462474999 65.7379967398 13.9449118000

H99 26.2213291428 65.4316345089 15.6657119998

H100 25.3211844967 63.1530000895 16.5759889407

H101 25.9982655880 63.8784379171 12.3974652934

H102 25.8413670753 61.4414349836 11.9500263173

H103 25.1971046446 60.7215991638 16.1339087817

H104 32.7367308604 55.7382055971 23.1667361877

H105 31.0126963148 55.6054448672 22.8206470869

H106 31.9546814796 56.9430196229 22.1356566737

H107 33.3342711089 55.2670689853 20.8168830906

H108 32.3374715347 53.9627777653 21.4342653797

H109 31.3815313749 56.2606449112 19.6465366618

H110 31.7396915778 54.6195289701 19.1119299281

H111 29.9228298287 53.7734089341 20.7107637199

H112 29.4620832576 55.4389055441 21.0063462502

H113 29.3875094189 54.0043709024 18.2875009295

H114 28.0040667401 54.3392201020 19.3317607679

H115 28.6067219945 56.7240124979 19.0633888849

H116 29.4438075177 62.3194470634 17.7612027165

H117 28.3132370224 62.3615145516 16.3942626134

H118 29.8697949746 61.5413759652 16.2399915096

H119 27.0244072467 60.8292470845 20.5509124569

H120 28.5882775099 61.4658682760 20.1167252090

H121 26.8913032959 63.2702625971 20.2366622955

H122 25.9124124357 62.4829035974 18.9893050148

H123 27.4958835884 63.1553701482 18.5822462534

H124 31.0226758942 59.2462362166 13.5560703957

H125 31.8244067026 57.9331743108 12.7280124111

H126 29.0238885689 59.0066668246 12.0080207740

H127 29.8590027665 57.6908432220 11.1618383975

H128 30.5176299784 59.3330719499 11.1118171339

H129 29.5974428587 59.7142758592 15.0780382790

H130 25.9497690462 59.0527275167 20.2824300104

H131 29.4302157079 53.3701766003 14.2814882532

H132 29.2352727056 50.9563599368 15.2664293150

H133 28.0155585008 51.1947545697 14.0140403948

H134 27.6991757053 50.0963261646 15.3633368924

H135 26.2679975714 50.0336123242 17.1208545723

H136 25.4551543043 51.0450791421 18.2949735024

H137 23.7841164156 50.0463061788 16.7854973544

H138 23.8281699386 51.8090999590 16.5009001203

H139 24.6454304309 53.0826323223 18.4989006604

H140 31.5269054589 55.7282097239 12.1178105366

H141 30.0806289751 54.7351042299 11.9316603871

H142 31.3286793490 54.2715803673 13.0921759993

H143 24.6804753486 57.9659795512 21.9809430071

H144 23.3822186736 56.7745840180 22.0203938692

H145 23.2416719026 58.2033081863 20.9896810514

H146 23.4160645896 53.4737952297 20.2046497268

H147 23.2485436063 54.6251452540 21.5031813130

H148 21.0681379830 54.4085516088 20.6938259787

H149 21.5864796256 55.9391696960 19.9719370340

H150 25.2322881151 49.2310893357 13.8046146674

H151 21.1551133741 52.7063196916 17.6821871407

H152 29.6558738998 56.2994256080 17.8903092349

H153 22.7638971743 60.5835864178 16.1810555025

H154 24.5689097538 59.2405585452 14.2806973116

N155 26.3048435237 56.8091217806 15.7584905083

O156 25.7884203641 57.2521462869 14.8502357756

The doublet NO structure after the first reduction.

Energies: E= -3397.226347, solv = -0.144344, disp = -180.93 Z_0_ = 844.68

Eps=20: solv=-0.188950

Fe1 27.3157386252 56.3208951141 16.9543772450

C2 25.3119296611 53.8392581575 18.1636879630

C3 28.8899793901 54.0822514023 14.9087853801

C4 29.0737820270 58.8831012532 15.5553253814

C5 26.2170823120 58.4086561164 19.4306956538

C6 26.2973168168 53.4794845602 17.2482882195

C7 26.5366212400 52.1213087914 16.8055421506

C8 27.5570145699 52.1740731003 15.8883866135

C9 27.9392204909 53.5742428864 15.7952152766

C10 28.1569780996# 51.0510113507# 15.0929936433#

C11 25.7165083796 50.9441172661 17.2373070688

C12 24.3966052250 50.8601123124 16.4227536480

C13 24.7229798775# 50.6400113384# 14.9749945299#

O14 25.0511908759 49.3625011510 14.7401192287

O15 24.7143753279 51.4828438600 14.0820849253

C16 29.2168323436 55.4281593511 14.7508498921

C17 30.1005994226 55.9467821064 13.7205302609

C18 30.1339917351 57.3073086004 13.8885070109

C19 29.2723853361 57.6094263224 15.0237504851

C20 30.7959742116# 55.1300165086# 12.6669939201#

C21 30.8323702510 58.3054577658 13.0134648225

C22 30.0089716850# 58.6030164330# 11.7499929289#

C23 28.3441439973 59.1892264741 16.7043130116

C24 28.3655519773 60.4750848161 17.3930567996

C25 27.6649151934 60.2947720854 18.5613012787

C26 27.1323153343 58.9377197389 18.5202789850

C27 29.0409714997# 61.7380135978# 16.9269939832#

C28 27.5503657167 61.2637956753 19.7086414801

C29 26.9359740382# 62.6240158223# 19.3539939933#

C30 25.6144453001 57.1507059921 19.3560794227

C31 24.5491285815 56.6805186743 20.2288498169

C32 24.2380981613 55.4064760198 19.8203102261

C33 25.1548151381 55.0965017107 18.7368032391

C34 23.9419773469# 57.4440116603# 21.3619972848#

C35 23.2053603909 54.4896572676 20.4231638196

C36 21.7278485647 54.7767857978 20.0104872422

C37 21.3749771751# 54.3240100851# 18.6159944215#

O38 21.2496364574 52.9921452891 18.5257378612

O39 21.2213190877 55.0433022346 17.6349660405

N40 27.1733074053 54.3370391872 16.6385706045

N41 28.7300715109 56.4516456047 15.5255257337

N42 27.5871600921 58.2818388038 17.4015269292

N43 25.9510887276 56.1761491308 18.4550977218

C44 31.9819766619# 55.8750149869# 22.3859916182#

C45 32.3351848963 54.9677871258 21.2007824970

C46 31.3933450832 55.1085316736 19.9922862231

C47 29.9387789200 54.7182407858 20.2922290034

C48 29.0131924225 54.6840006186 19.0712142084

N49 28.8268018998 55.9966224259 18.4025307192

C50 30.4419719136# 51.7440156054# 8.3349940421#

C51 29.4598396799 52.7775621079 8.9018185727

C52 27.9945238774 52.3297324134 8.7951529396

C53 26.9751900606 53.3999351667 9.2116577622

N54 26.9835171106 53.7458065359 10.6463298682

C55 26.2648280613 53.1456270638 11.6067919088

N56 25.4967137257 52.0783127307 11.3429446206

N57 26.2755452439 53.6195884599 12.8585250107

C58 24.1709721262# 65.7240050282# 15.0359965976#

C59 25.5437467306 65.1712541015 14.6145442856

C60 25.4954405939 63.6885628409 14.3150470095

C61 25.6336938298 62.7420463016 15.3420507569

C62 25.2634956424 63.2165198016 13.0160940821

C63 25.5473916774 61.3709263374 15.0928593745

C64 25.1635221537 61.8502976558 12.7455180479

C65 25.3098638735 60.9375582179 13.7882791885

O66 25.2078926109 59.5615844323 13.5740432730

C67 19.2469763647# 58.8630055827# 17.5249980372#

C68 20.6507126646 59.3548285945 17.9151897063

C69 21.7051601736 58.9075040222 16.9537580730

N70 22.0175907075 57.5685640228 16.7481344607

C71 22.5083292141 59.6098314318 16.0964954493

C72 22.9660095433 57.4658584588 15.8152751597

N73 23.2759111960 58.6930207746 15.4014188968

H74 25.0058627319 51.6356578572 12.1173227189

H75 25.4402891991 51.6781073355 10.4201163905

H76 26.9322827955 54.3260864726 13.1690528034

H77 25.7852443344 53.1050476401 13.5868230196

H78 27.5359206648 54.5436666027 10.9299066822

H79 29.7087843246 52.9823435966 9.9530117137

H80 29.5816598664 53.7279238593 8.3625765967

H81 27.7706333536 52.0668075552 7.7526561236

H82 27.8367189863 51.4143855650 9.3829349449

H83 27.1706794504 54.3332250821 8.6749807135

H84 25.9547664766 53.1071850492 8.9386257425

H85 31.4749045913 52.0931572627 8.4282456236

H86 30.2475738782 51.5578489508 7.2728780519

H87 30.3638014228 50.7869252711 8.8637204461

H88 18.9613078429 59.2337735626 16.5358154976

H89 18.5080948643 59.2205551271 18.2480292291

H90 19.1935844501 57.7687109663 17.5081162736

H91 20.6708478587 60.4485770070 17.9543606768

H92 20.9038083668 59.0053029926 18.9250786549

H93 21.6190815289 56.7463548713 17.2292357873

H94 23.4124402668 56.5513982161 15.4629638628

H95 24.2329437766 66.7955694327 15.2528358765

H96 23.8022437935 65.2178848438 15.9355790046

H97 23.4289217753 65.5819927131 14.2424363760

H98 25.9011562873 65.7140063757 13.7315084556

H99 26.2728244075 65.3622024354 15.4116899588

H100 25.8222109614 63.0800941078 16.3579860102

H101 25.1567906040 63.9246386180 12.1986986013

H102 24.9785923566 61.5062474012 11.7296145252

H103 25.7021001702 60.6493273117 15.8893757045

H104 32.7282545798 55.7834281478 23.1820050766

H105 31.0088298655 55.6230948738 22.8222094128

H106 31.9515437015 56.9292027497 22.0815740354

H107 33.3586862687 55.1874745553 20.8717697183

H108 32.3402120735 53.9192636210 21.5297794317

H109 31.4364635646 56.1470727853 19.6297205593

H110 31.7687627296 54.4786279746 19.1721451006

H111 29.9213940288 53.7178322786 20.7476745186

H112 29.5107692112 55.3976210242 21.0427806701

H113 29.3922558104 53.9872617282 18.3170904757

H114 28.0208537173 54.3337883016 19.3667630462

H115 28.6200608319 56.7040691197 19.1092595670

H116 29.4313548362 62.3133577139 17.7714503884

H117 28.3483800946 62.3800362174 16.3703066920

H118 29.8870922520 61.5261657151 16.2653234362

H119 26.9771532931 60.8146159431 20.5267322399

H120 28.5568111103 61.4293069811 20.1196928899

H121 26.8860559488 63.2633596703 20.2417125046

H122 25.9148222747 62.5051018607 18.9719575122

H123 27.5179972604 63.1558801163 18.5982523476

H124 31.0221430130 59.2363834259 13.5582629620

H125 31.8190797398 57.9235822901 12.7249532655

H126 29.0251779898 59.0083189693 12.0131588289

H127 29.8535076892 57.6920088117 11.1611384201

H128 30.5158342022 59.3336628720 11.1100732452

H129 29.5938282770 59.7022739724 15.0731081864

H130 25.9043774738 59.0512751480 20.2458529079

H131 29.4029538967 53.3698583433 14.2703817585

H132 29.2426105750 50.9886935054 15.2336544625

H133 27.9826518685 51.1805836154 14.0162017299

H134 27.7365945093 50.0841623468 15.3824434268

H135 26.2703329378 50.0084045443 17.1154468645

H136 25.4588723573 51.0119310087 18.2989692100

H137 23.7864495082 50.0231014601 16.7769201627

H138 23.8321880146 51.7904251465 16.5149797564

H139 24.6631873000 53.0501457441 18.5255979167

H140 31.5084156093 55.7369733841 12.1025953482

H141 30.0898042517 54.6982928006 11.9425307688

H142 31.3531685115 54.2922504162 13.1030560018

H143 24.7098570839 57.8791972645 22.0125346918

H144 23.3153050470 56.8023937169 21.9881745159

H145 23.3148990544 58.2762574706 21.0137457340

H146 23.4325494933 53.4407221610 20.2095505696

H147 23.2396665082 54.5810145541 21.5145368745

H148 21.0665257989 54.2470245200 20.7045601068

H149 21.5171023002 55.8461499772 20.0835973505

H150 25.2830137946 49.2571267634 13.7983032678

H151 20.9877911843 52.7819285952 17.6081609070

H152 29.6969579020 56.2923092344 17.9594249790

H153 22.6016843638 60.6705149671 15.9269619800

H154 23.9980726241 58.9170281461 14.6964776786

H155 25.1061436142 59.3977858186 12.6223560649

N156 26.0814135345 56.5926032065 15.7140731199

O158 25.7915620116 56.2382403904 14.6183227791

The singlet NOH structure after the second reduction.

Energies: E= -3397.812024, solv = -0.141900, disp = -185.01 Z_0_ = 852.28

Eps=20: solv=-0.185802

Fe1 27.3287889347 56.3231659349 16.9325244600

C2 25.2802155416 53.8701970541 18.0973364071

C3 28.9517465553 54.0734192194 14.9420607671

C4 29.0747172330 58.8809499068 15.5542811199

C5 26.1936240049 58.4200782699 19.4134364660

C6 26.2827198100 53.5111532880 17.2009897926

C7 26.5338507861 52.1470497314 16.7843929760

C8 27.5740824848 52.1846127503 15.8887567728

C9 27.9683818155 53.5793696967 15.7928259160

C10 28.1569783455# 51.0510109636# 15.0929934777#

C11 25.7136698093 50.9737476751 17.2292238923

C12 24.3931681552 50.8765747890 16.4195519373

C13 24.7229794533# 50.6400111408# 14.9749948296#

O14 25.0412273954 49.3563371319 14.7539836640

O15 24.7282039424 51.4728626575 14.0752663116

C16 29.2650882603 55.4200324447 14.7806495054

C17 30.1241677093 55.9437026114 13.7374058360

C18 30.1426526110 57.3067690156 13.8969403665

C19 29.2878710840 57.6043346946 15.0370881938

C20 30.7959744253# 55.1300164026# 12.6669936721#

C21 30.8304006451 58.3081480472 13.0158506991

C22 30.0089716947# 58.6030164302# 11.7499929306#

C23 28.3363447065 59.1903294131 16.6957823863

C24 28.3587534871 60.4748761385 17.3848139892

C25 27.6554010986 60.2948933531 18.5531653616

C26 27.1168753882 58.9440742017 18.5083260228

C27 29.0409715390# 61.7380137053# 16.9269939879#

C28 27.5440690230 61.2602843358 19.7036317867

C29 26.9359740207# 62.6240158780# 19.3539940099#

C30 25.5778062004 57.1724064499 19.3178193967

C31 24.5229788460 56.6924135400 20.1971753221

C32 24.2109875460 55.4193964743 19.7742139831

C33 25.1141948226 55.1252560566 18.6731581172

C34 23.9419772430# 57.4440117583# 21.3619975147#

C35 23.2330343391 54.4561042299 20.4019058728

C36 21.7262430679 54.6491054727 20.0477469905

C37 21.3749769370# 54.3240099074# 18.6159945200#

O38 21.2217825769 53.0110580903 18.3974381444

O39 21.2561156583 55.1442771772 17.7130222814

N40 27.1736400007 54.3608887542 16.5995295456

N41 28.7691955921 56.4412032214 15.5535735503

N42 27.5734269092 58.2846864981 17.3911316789

N43 25.9051020038 56.2097921938 18.3907815745

C44 31.9819767183# 55.8750149950# 22.3859916866#

C45 32.3806637094 55.1485758144 21.0952221169

C46 31.3912650958 55.3235339238 19.9293257868

C47 29.9924221220 54.7504733710 20.2053665816

C48 29.0631644129 54.7063961418 18.9868312501

N49 28.7651004468 56.0437758834 18.4063070528

C50 30.4419722378# 51.7440155261# 8.3349937363#

C51 29.6518868911 52.8459718540 9.0601670797

C52 28.1326715470 52.6400252465 8.9664184473

C53 27.2630851927 53.7899336548 9.5067423713

N54 27.2416485146 53.9700126917 10.9741194607

C55 26.3930994335 53.3407325640 11.8064457575

N56 25.7123120137 52.2578015363 11.3735746143

N57 26.1809855892 53.7729634355 13.0459854986

C58 24.1709718679# 65.7240055974# 15.0359966735#

C59 25.3288473352 65.2069456301 14.1644385546

C60 25.2892006816 63.7045028352 13.9831466558

C61 25.6634289644 62.8499119775 15.0324398197

C62 24.8427003615 63.1189396901 12.7919243036

C63 25.5966571869 61.4622788594 14.9086763802

C64 24.7652629660 61.7315283514 12.6463516408

C65 25.1418656753 60.9130099004 13.7085610335

O66 25.0636034524 59.5235430294 13.6271355434

C67 19.2469765174# 58.8630056947# 17.5249979603#

C68 20.5540511804 59.5008150278 18.0209842830

C69 21.7505547864 59.0553180880 17.2426457988

N70 22.1993306996 57.7386656094 17.2302700568

C71 22.6030735587 59.7420792885 16.4215115429

C72 23.2792598331 57.6340218921 16.4538935166

N73 23.5353651936 58.8376731719 15.9455166419

H74 25.1515868244 51.7579387952 12.0585653957

H75 26.0558891922 51.7353837200 10.5810795820

H76 26.4681252533 54.6868221809 13.4270926393

H77 25.6481707732 53.1798048861 13.6785416639

H78 27.7792035650 54.7277724275 11.3739479345

H79 29.9579523722 52.8888302875 10.1147609857

H80 29.9057231208 53.8219386408 8.6228039508

H81 27.8547725579 52.5187039025 7.9107237765

H82 27.8627499091 51.6916210188 9.4555304762

H83 27.6086712049 54.7398946321 9.0882563079

H84 26.2250252357 53.6627089229 9.1800112894

H85 31.5192561205 51.9170880060 8.4179289457

H86 30.1885503710 51.7150514517 7.2694302080

H87 30.2291934925 50.7553649918 8.7584517756

H88 19.0534541269 59.1255768581 16.4803598169

H89 18.4040117340 59.2164432531 18.1256605792

H90 19.2758312292 57.7702358148 17.6018750176

H91 20.4923101335 60.5915087940 17.9506004984

H92 20.7047076685 59.2692267849 19.0838110605

H93 21.7588922550 56.9109801861 17.6573229946

H94 23.8754728705 56.7508543233 16.2933875691

H95 24.2330600979 66.8102066013 15.1589787021

H96 24.1933429179 65.2711251415 16.0338809942

H97 23.2020139339 65.4895644683 14.5815558452

H98 25.2969221203 65.6959980474 13.1841342728

H99 26.2831523047 65.4961796720 14.6230018390

H100 26.0169525228 63.2770337240 15.9679196623

H101 24.5494542735 63.7526777287 11.9593971166

H102 24.4149499748 61.2995062443 11.7107380436

H103 25.9241249918 60.8166387597 15.7185093894

H104 32.7590545824 55.7634827326 23.1489934240

H105 31.0514985455 55.4803208188 22.8095977328

H106 31.8429851146 56.9491006985 22.2093229788

H107 33.3644951666 55.5091506584 20.7697293112

H108 32.5031892513 54.0759444919 21.3003053720

H109 31.3169949035 56.3949481440 19.6871747589

H110 31.8063081327 54.8324188340 19.0366578335

H111 30.0907039179 53.7204426287 20.5763306231

H112 29.5012423190 55.3148535769 21.0104908047

H113 29.5013421250 54.1012390421 18.1880122932

H114 28.1065591461 54.2519947030 19.2560724974

H115 28.4784338544 56.6779491192 19.1536552950

H116 29.4339329351 62.3048882314 17.7756563391

H117 28.3518149404 62.3882368982 16.3755660105

H118 29.8846029075 61.5264778522 16.2624562795

H119 26.9682493504 60.8100265690 20.5192316452

H120 28.5510598420 61.4190471303 20.1161023160

H121 26.8761259696 63.2546488066 20.2471763714

H122 25.9202355656 62.5110151518 18.9566767073

H123 27.5286882683 63.1624239979 18.6111735428

H124 31.0150245067 59.2408677816 13.5594004412

H125 31.8197284343 57.9316979843 12.7294027443

H126 29.0223739402 59.0034255264 12.0091495905

H127 29.8604290732 57.6931252098 11.1578316770

H128 30.5160753162 59.3366710679 11.1140751507

H129 29.5895369476 59.6999755677 15.0665934395

H130 25.8867001979 59.0570706047 20.2348040221

H131 29.4736196238 53.3602001934 14.3130704238

H132 29.2434039040 50.9785646890 15.2203111651

H133 27.9680586423 51.1828866984 14.0194699952

H134 27.7293539018 50.0902029357 15.3909025988

H135 26.2704487711 50.0388242504 17.1159028483

H136 25.4617227165 51.0523742762 18.2916447974

H137 23.7851677346 50.0430873843 16.7849982958

H138 23.8254600466 51.8059771321 16.4997476500

H139 24.6333153907 53.0771996107 18.4518310092

H140 31.4340694687 55.7543896243 12.0365837857

H141 30.0706678463 54.6306561039 12.0098304292

H142 31.4287551515 54.3403796153 13.0911351761

H143 24.7277032035 57.8261793328 22.0240993275

H144 23.2921143730 56.8035671265 21.9649652741

H145 23.3472067243 58.3090251769 21.0401389914

H146 23.5122110846 53.4221133645 20.1769367817

H147 23.2977236317 54.5448058862 21.4923139131

H148 21.1357554578 53.9986579503 20.7007357739

H149 21.4290943997 55.6842230406 20.2347952827

H150 25.2649177385 49.2420322151 13.8110625965

H151 20.9704072637 52.8953568249 17.4600476317

N152 26.0879429386 56.3278683217 14.4219655601

O153 26.0692941187 56.8047233408 15.6438912288

H154 29.6152100162 56.4444893773 18.0087105785

H155 22.6308786933 60.7839093236 16.1450316281

H156 25.4206209407 56.9350014071 13.9129722297

H157 24.2619493776 59.0301413030 15.2465402648

H158 24.6731191844 59.2912937849 12.7688514906

The doublet ONH_2_ structure after the third reduction.

Energies: E= -3398.439331, solv = -0.141590, disp = -187.10 Z_0_ = 860.92

Eps=20: solv=-0.185617

Fe1 27.2929426735 56.3270691126 16.9093886132

C2 25.2804203667 53.8675647266 18.1011279697

C3 28.9510869062 54.0757305200 14.9414191691

C4 29.0850142303 58.8866441768 15.5512368354

C5 26.1615150208 58.4248747362 19.3826944921

C6 26.2875625774 53.5102154298 17.2082793274

C7 26.5358501070 52.1467277428 16.7856734909

C8 27.5729068759 52.1857603470 15.8866356696

C9 27.9689354169 53.5810451249 15.7939699891

C10 28.1569783479# 51.0510109406# 15.0929934520#

C11 25.7209400709 50.9694680979 17.2306482738

C12 24.3999109678 50.8607121702 16.4241448609

C13 24.7229794481# 50.6400111413# 14.9749948358#

O14 25.0780567903 49.3679201789 14.7435731940

O15 24.6884349059 51.4780829425 14.0815322454

C16 29.2729860171 55.4208051734 14.7814462604

C17 30.1320265769 55.9435832018 13.7393011237

C18 30.1561014122 57.3081342826 13.8967275100

C19 29.3051330579 57.6100772260 15.0360459870

C20 30.7959744267# 55.1300164047# 12.6669936645#

C21 30.8401698557 58.3074850987 13.0098049469

C22 30.0089716941# 58.6030164337# 11.7499929275#

C23 28.3335416885 59.1925227946 16.6847411873

C24 28.3504791236 60.4752386763 17.3754817911

C25 27.6353009235 60.2931017239 18.5371171101

C26 27.0922413934 58.9450407183 18.4828424005

C27 29.0409715430# 61.7380137140# 16.9269939879#

C28 27.5244003071 61.2499640668 19.6947391768

C29 26.9359740191# 62.6240158858# 19.3539940119#

C30 25.5463479762 57.1766992654 19.2925684768

C31 24.5041131612 56.6926450721 20.1883184056

C32 24.1969725665 55.4177207317 19.7728417468

C33 25.0961191823 55.1258481866 18.6641896103

C34 23.9419772393# 57.4440117656# 21.3619975350#

C35 23.2250996147 54.4534090612 20.4061984151

C36 21.7193317970 54.6463816686 20.0499835476

C37 21.3749768947# 54.3240098477# 18.6159945534#

O38 21.2115758469 53.0115219258 18.3985665859

O39 21.2695857860 55.1430357225 17.7106908649

N40 27.1830228416 54.3573461897 16.6113228104

N41 28.7689636093 56.4477041176 15.5465197574

N42 27.5613061525 58.2854595771 17.3700383793

N43 25.8699562268 56.2153254835 18.3679686875

C44 31.9819767203# 55.8750149948# 22.3859916862#

C45 32.2977070580 55.0218125486 21.1507293852

C46 31.3220883494 55.2080663575 19.9744559995

C47 29.8799152908 54.7773463096 20.2852553583

C48 28.9452701631 54.7249129639 19.0706128503

N49 28.7172752602 56.0426357293 18.4196735529

C50 30.4419722505# 51.7440155179# 8.3349937386#

C51 29.6306699151 52.8366667986 9.0549600149

C52 28.1165411400 52.5743695636 9.0146738917

C53 27.2119879277 53.7169503414 9.5177065951

N54 27.1696809824 53.9496101374 10.9791073686

C55 26.3037855742 53.3496682140 11.8202942892

N56 25.6762999667 52.2215284133 11.4241057953

N57 26.0331873733 53.8488989259 13.0236596428

C58 24.1709718538# 65.7240056399# 15.0359966696#

C59 25.4226880228 65.2489097831 14.2771490692

C60 25.4012802168 63.7546463057 14.0429265342

C61 25.7268299892 62.8660681508 15.0793691077

C62 24.9908846379 63.2113389441 12.8188824838

C63 25.6347851617 61.4832930651 14.9169606044

C64 24.8885170137 61.8308589076 12.6348211723

C65 25.2031052200 60.9778141551 13.6903886131

O66 25.0734787305 59.5919956962 13.5664068713

C67 19.2469765624# 58.8630057133# 17.5249979368#

C68 20.5703737642 59.4602785322 18.0295536285

C69 21.7578996922 59.0410300692 17.2214160179

N70 22.2382642906 57.7345654208 17.1895959501

C71 22.5817320811 59.7560498422 16.3960199104

C72 23.3088861935 57.6601888607 16.3942212222

N73 23.5289460933 58.8780071524 15.9001379441

H74 25.1051570294 51.7420016210 12.1159190029

H75 26.0751624411 51.6660130433 10.6814195361

H76 26.2279071177 54.8178855941 13.3552573570

H77 25.5072572903 53.2625832677 13.6675890214

H78 27.6735464134 54.7423999225 11.3523903999

H79 29.9675841535 52.9224342983 10.0973955896

H80 29.8342514155 53.8077752362 8.5818977543

H81 27.8186486930 52.3922207491 7.9731554350

H82 27.8937282202 51.6413114923 9.5541595871

H83 27.5349862795 54.6611566063 9.0690568807

H84 26.1810234593 53.5494252355 9.1867698136

H85 31.5136468486 51.9598761076 8.3819350148

H86 30.1591146210 51.6774277101 7.2786007437

H87 30.2791050399 50.7599599348 8.7900183859

H88 19.0430106047 59.1739246391 16.4957555804

H89 18.4180940498 59.2018104481 18.1531020998

H90 19.2622993225 57.7676843225 17.5535118081

H91 20.5198966269 60.5537525301 18.0088767793

H92 20.7303629999 59.1806790127 19.0793753179

H93 21.8175724457 56.8986965739 17.6203425810

H94 23.9479241266 56.8063987364 16.2071192633

H95 27.1919552589 56.8901099243 14.1174066269

H96 24.2050284399 66.8053161329 15.2048441404

H97 24.0916909773 65.2327134634 16.0125161039

H98 23.2603261036 65.4981975777 14.4703844399

H99 25.4917680600 65.7734390150 13.3172343777

H100 26.3195175080 65.5229360765 14.8468102490

H101 26.0513248631 63.2620981944 16.0385955553

H102 24.7394754505 63.8732209052 11.9946325316

H103 24.5622990499 61.4299548218 11.6769141644

H104 25.9140407020 60.8105397839 15.7226162011

H105 32.7459543617 55.7371506359 23.1578625114

H106 31.0168118313 55.6109217428 22.8328697467

H107 31.9554903988 56.9425839041 22.1333848589

H108 33.3102970581 55.2585232689 20.8005809503

H109 32.3149617012 53.9598844622 21.4329386063

H110 31.3411367215 56.2636598444 19.6623133666

H111 31.6884182274 54.6248365832 19.1164849072

H112 29.8931682618 53.7706087180 20.7263168232

H113 29.4408419023 55.4345692055 21.0489848177

H114 29.3431187375 54.0515798972 18.3059683090

H115 27.9651298970 54.3422753046 19.3657809557

H116 28.4560596810 56.7242822414 19.1333132378

H117 29.4367988955 62.2961530498 17.7802484869

H118 28.3566032207 62.3963704807 16.3799522720

H119 29.8843005841 61.5263512454 16.2621829333

H120 26.9365818077 60.7992342868 20.5012978135

H121 28.5298795901 61.3922761267 20.1169198647

H122 26.8748508531 63.2455468933 20.2534060034

H123 25.9227404507 62.5274408378 18.9461469982

H124 27.5420641203 63.1637177496 18.6230143294

H125 31.0299707404 59.2403365204 13.5513051396

H126 31.8263164601 57.9293191199 12.7154368535

H127 29.0248709538 59.0054284849 12.0167507212

H128 29.8562027935 57.6932239201 11.1588131065

H129 30.5110439037 59.3363638998 11.1100672130

H130 29.6052174706 59.7064413601 15.0705196011

H131 25.8539776201 59.0646993439 20.2015178444

H132 29.4691139296 53.3616985093 14.3102328687

H133 29.2455597855 50.9900479750 15.2075809458

H134 27.9534318093 51.1722225409 14.0206670657

H135 27.7419762667 50.0887306751 15.4034736125

H136 26.2841078078 50.0383642346 17.1172214373

H137 25.4697101955 51.0465645323 18.2933827323

H138 23.8056474398 50.0159672288 16.7865612840

H139 23.8184573578 51.7807604560 16.5135671273

H140 24.6403122150 53.0702558517 18.4585254955

H141 31.4332844488 55.7518686331 12.0332797972

H142 30.0648547786 54.6329622063 12.0146911900

H143 31.4286692099 54.3386892721 13.0881142013

H144 24.7385209075 57.8213021459 22.0139626403

H145 23.2974168525 56.8051558806 21.9723448977

H146 23.3462158664 58.3118065034 21.0497568510

H147 23.5052923682 53.4193501701 20.1825734642

H148 23.2903702532 54.5450721771 21.4963321171

H149 21.1270651823 53.9948579016 20.7001763562

H150 21.4222650839 55.6814953237 20.2371521155

H151 25.2905530548 49.2627267405 13.7971659960

H152 20.9671292161 52.8974335121 17.4591365411

N153 26.2492764304 56.5186282761 14.2533711294

O154 25.9815893521 56.6857072129 15.6589363293

H155 29.5876347104 56.3834838934 18.0098962581

H156 22.5809917205 60.8016276107 16.1327168433

H157 25.6438490654 57.2274968783 13.8312522446

H158 24.2531613744 59.0920895676 15.2082310319

H159 24.6200086181 59.4095765055 12.7268097216

The singlet NH_2_OH structure after the fourth reduction.

Energies: E= -3399.033438, solv = -0.142930, disp = -186.01 Z_0_ = 868.32

Fe1 27.3577185678 56.3046745445 16.9775506686

C2 25.2754105793 53.8529257320 18.1107072448

C3 28.9231566342 54.0799582286 14.9335715226

C4 29.0926922172 58.8921807307 15.5418033236

C5 26.1210238368 58.4330861616 19.3407619754

C6 26.2724585488 53.4945052095 17.2054007791

C7 26.5285160089 52.1279393926 16.7847834622

C8 27.5574909431 52.1774882332 15.8813639120

C9 27.9382511304 53.5808582236 15.7834157563

C10 28.1569782482# 51.0510111240# 15.0929935404#

C11 25.7231913332 50.9449761561 17.2287504316

C12 24.3976938365 50.8326016287 16.4268049824

C13 24.7229795046# 50.6400112723# 14.9749948140#

O14 25.0159664619 49.3601520886 14.7047135309

O15 24.7515173971 51.5127118306 14.1131214889

C16 29.2666854490 55.4226001517 14.7797502020

C17 30.1329484570 55.9413632467 13.7376130036

C18 30.1694413774 57.3044390559 13.8957329993

C19 29.3234490612 57.6098674876 15.0395164100

C20 30.7959743628# 55.1300164757# 12.6669937758#

C21 30.8506967358 58.2999455586 13.0014785792

C22 30.0089716809# 58.6030164699# 11.7499928636#

C23 28.3087181325 59.2136463086 16.6513610271

C24 28.3319071532 60.4837395363 17.3558541818

C25 27.6066228024 60.2980882513 18.5101930879

C26 27.0400738026 58.9639218372 18.4350179129

C27 29.0409715406# 61.7380137327# 16.9269939828#

C28 27.5089886736 61.2410471268 19.6796404465

C29 26.9359740157# 62.6240158966# 19.3539940186#

C30 25.5220141725 57.1748117031 19.2700005984

C31 24.4907748657 56.6912592850 20.1808996960

C32 24.1898271772 55.4118241778 19.7766808643

C33 25.0879263894 55.1153942168 18.6664109046

C34 23.9419772615# 57.4440117509# 21.3619974839#

C35 23.2164464339 54.4524364794 20.4136026445

C36 21.7124949243 54.6514355723 20.0492696377

C37 21.3749768431# 54.3240097703# 18.6159944620#

O38 21.2180964080 53.0114988114 18.4006846583

O39 21.2674452632 55.1389395920 17.7053007341

N40 27.1483745991 54.3542324721 16.5985812530

N41 28.7724626599 56.4512655485 15.5496140680

N42 27.4960852202 58.3085059119 17.3068758846

N43 25.8550020312 56.2079983726 18.3518995479

C44 31.9819766824# 55.8750149971# 22.3859916429#

C45 32.2881396732 54.9848903754 21.1749704673

C46 31.3173908469 55.1591822056 19.9936357752

C47 29.8670691060 54.7644574046 20.3134206164

C48 28.9276659874 54.7334535927 19.1024234461

N49 28.7277461633 56.0529684597 18.4481374505

C50 30.4419721305# 51.7440156067# 8.3349938315#

C51 29.5900601281 52.8528879953 8.9771963226

C52 28.0901843650 52.5184882011 8.9780726536

C53 27.1416248111 53.6520274657 9.4125022801

N54 27.1223696452 53.9972319149 10.8519251101

C55 26.3213389469 53.4158662929 11.7636708497

N56 25.7432931894 52.2324105436 11.4819993487

N57 26.0605693881 53.9968224744 12.9362075452

C58 24.1709719680# 65.7240052082# 15.0359966330#

C59 24.9813321340 65.2524540654 13.8142930009

C60 24.9311893092 63.7503556644 13.6307670982

C61 25.7435811509 62.9060991196 14.4044665975

C62 24.0490024177 63.1555522851 12.7192702667

C63 25.6826631104 61.5174694417 14.2855457763

C64 23.9665149912 61.7672677147 12.5866901630

C65 24.7855665690 60.9625458783 13.3744683902

O66 24.7178314300 59.5604763177 13.3031587908

C67 19.2469767614# 58.8630056950# 17.5249979505#

C68 20.5774049772 59.4355941600 18.0382948885

C69 21.7311598948 59.0183956163 17.1860141716

N70 22.1776472277 57.7036576710 17.1138801880

C71 22.5098497315 59.7228870819 16.3093989117

C72 23.1821743400 57.6119427158 16.2425847942

N73 23.3999637134 58.8288639690 15.7408338077

H74 25.2099195809 51.7818168948 12.2251433769

H75 26.1434978725 51.6440651043 10.7657319357

H76 26.2416822190 54.9870320801 13.1519456989

H77 25.6144700480 53.4402557441 13.6606075740

H78 27.6236955642 54.8211427277 11.1545308217

H79 29.9329878064 53.0360919673 10.0050117673

H80 29.7435849739 53.7917477535 8.4266089370

H81 27.7880380610 52.2486819635 7.9570403441

H82 27.9177762772 51.6175158257 9.5862403931

H83 27.4062762872 54.5728306846 8.8841645168

H84 26.1121584571 53.4108237676 9.1257548001

H85 31.5031801160 52.0103448119 8.3488569496

H86 30.1511019136 51.5805134724 7.2913835216

H87 30.3292664969 50.7932582367 8.8691345010

H88 19.0378610119 59.2081747853 16.5076820610

H89 18.4231164926 59.1854520566 18.1684850092

H90 19.2562062187 57.7673576801 17.5185327700

H91 20.5396206812 60.5294594707 18.0518714240

H92 20.7514588653 59.1186925119 19.0746449616

H93 21.7771686094 56.8704126664 17.5749192887

H94 23.7529422628 56.7287893264 16.0067214086

H95 27.3872046164 57.0048392043 13.9396108555

H96 24.2330227007 66.8117003347 15.1441837703

H97 24.5485690756 65.2707924980 15.9597503053

H98 23.1136795855 65.4544165026 14.9351958507

H99 24.6014802198 65.7445400356 12.9115888038

H100 26.0244690855 65.5732292163 13.9261129347

H101 26.4454760943 63.3440767836 15.1096136610

H102 23.4138088452 63.7818994682 12.0990284554

H103 23.2740123441 61.3266691106 11.8717515458

H104 26.3329567515 60.8777185006 14.8759659601

H105 32.7437206187 55.7503085837 23.1625074403

H106 31.0134677042 55.6348135948 22.8389740238

H107 31.9678187272 56.9354896486 22.1037636347

H108 33.3054110967 55.1965511373 20.8221365604

H109 32.2873403379 53.9304400921 21.4846134822

H110 31.3573912289 56.2062861275 19.6559412401

H111 31.6720227080 54.5485069847 19.1500592958

H112 29.8587633129 53.7604376670 20.7608479495

H113 29.4481275896 55.4370591378 21.0752537954

H114 29.3063625179 54.0492968448 18.3372087128

H115 27.9389775187 54.3744679365 19.3992932874

H116 28.4755170013 56.7395199745 19.1619483846

H117 29.4906197900 62.2532437707 17.7817088363

H118 28.3559960680 62.4456998316 16.4435276520

H119 29.8472638515 61.5314279253 16.2164867147

H120 26.9162268230 60.7870903356 20.4807349932

H121 28.5162005986 61.3670157973 20.1031281628

H122 26.8739611413 63.2340561707 20.2613305193

H123 25.9254535462 62.5430577174 18.9366027516

H124 27.5519458807 63.1684400914 18.6347879095

H125 31.0533984724 59.2313092917 13.5413665177

H126 31.8310054292 57.9163773592 12.6950597127

H127 29.0307377165 59.0142432182 12.0268718374

H128 29.8425372241 57.6944438795 11.1603411527

H129 30.5075295841 59.3339074043 11.1043243981

H130 29.6346742035 59.7047422574 15.0716943637

H131 25.8173560797 59.0710547576 20.1631087144

H132 29.4409597654 53.3644766750 14.3025686006

H133 29.2445891332 50.9951976557 15.2213814037

H134 27.9690803802 51.1713752122 14.0171526433

H135 27.7445916923 50.0842701083 15.3945156499

H136 26.2895419224 50.0162500531 17.1094412711

H137 25.4727629001 51.0160356830 18.2923176490

H138 23.8120716652 49.9784343165 16.7799029762

H139 23.8116621341 51.7481202666 16.5333314201

H140 24.6434996623 53.0544937299 18.4827507543

H141 31.4347766885 55.7507588352 12.0334406953

H142 30.0654920505 54.6323149266 12.0132614288

H143 31.4274631212 54.3373595846 13.0878300525

H144 24.7474593501 57.8090895238 22.0101788222

H145 23.2945511727 56.8093941358 21.9739269632

H146 23.3542116997 58.3209628947 21.0595713447

H147 23.4924756333 53.4161080501 20.1953464794

H148 23.2757871960 54.5486578483 21.5036739510

H149 21.1123349432 54.0054989040 20.6980027099

H150 21.4207623275 55.6890608019 20.2305327110

H151 25.2454267681 49.2759347434 13.7603919838

H152 20.9804322540 52.8924592389 17.4602355960

N153 26.3716634220 56.9011280371 13.8888512332

O154 25.9246658510 57.0099978436 15.2636857098

H155 29.6103826424 56.3762445875 18.0501195974

H156 22.5081638909 60.7690948754 16.0485234353

H157 26.0022121640 57.7378223396 13.4285542708

H158 24.0477228442 59.0354332002 14.9733123433

H159 24.0981860127 59.3235841514 12.5921122406

H160 26.2912526751 57.8431863974 15.6454146486

The TS for the cleavage of the second N-O bond in **Figure 5.**

Energies: E= -3399.013477, solv = -0.143760, disp = -186.32 Z_0_ = 865.40

Fe1 27.3794000000 56.3162130000 16.9916620000

C2 25.3343860000 53.8512010000 18.1588880000

C3 28.9229270000 54.0771070000 14.9236180000

C4 29.1227840000 58.8791030000 15.5920200000

C5 26.2325570000 58.4145590000 19.4440260000

C6 26.3072460000 53.4914760000 17.2326920000

C7 26.5431700000 52.1274650000 16.7991830000

C8 27.5658130000 52.1746300000 15.8886800000

C9 27.9538200000 53.5743710000 15.7950610000

C10 28.1569780000 51.0510110000 15.0929940000

C11 25.7246130000 50.9500510000 17.2324940000

C12 24.4000830000 50.8601410000 16.4235430000

C13 24.7229800000 50.6400110000 14.9749950000

O14 25.0007590000 49.3544980000 14.7241080000

O15 24.7614740000 51.4982900000 14.0972420000

C16 29.2567560000 55.4229440000 14.7760190000

C17 30.1216180000 55.9456190000 13.7358420000

C18 30.1580900000 57.3080650000 13.9044240000

C19 29.3254020000 57.6069880000 15.0605130000

C20 30.7959740000 55.1300160000 12.6669940000

C21 30.8381040000 58.3079840000 13.0137530000

C22 30.0089720000 58.6030160000 11.7499930000

C23 28.3713170000 59.1879110000 16.7266930000

C24 28.3824060000 60.4753150000 17.4088850000

C25 27.6801210000 60.2981210000 18.5753590000

C26 27.1475570000 58.9438090000 18.5351580000

C27 29.0409720000 61.7380140000 16.9269940000

C28 27.5562480000 61.2701930000 19.7178110000

C29 26.9359740000 62.6240160000 19.3539940000

C30 25.6321320000 57.1556890000 19.3668480000

C31 24.5593440000 56.6836070000 20.2298450000

C32 24.2522990000 55.4120910000 19.8127720000

C33 25.1778080000 55.1062420000 18.7344720000

C34 23.9419770000 57.4440120000 21.3619970000

C35 23.2220730000 54.4882310000 20.4107490000

C36 21.7386300000 54.7687140000 20.0118560000

C37 21.3749770000 54.3240100000 18.6159950000

O38 21.1880890000 52.9992470000 18.5290410000

O39 21.2685020000 55.0472910000 17.6333820000

N40 27.1823330000 54.3458770000 16.6196260000

N41 28.7753800000 56.4450170000 15.5637950000

N42 27.6038740000 58.2810590000 17.4156960000

N43 25.9781660000 56.1853720000 18.4633810000

C44 31.9819770000 55.8750150000 22.3859920000

C45 32.3670290000 55.0170670000 21.1746070000

C46 31.4109860000 55.1450640000 19.9757360000

C47 29.9809420000 54.6642790000 20.2652220000

C48 29.0493210000 54.6403170000 19.0481330000

N49 28.8007030000 55.9787090000 18.4506120000

C50 30.4419720000 51.7440160000 8.3349940000

C51 29.4887070000 52.7943890000 8.9204530000

C52 28.0115520000 52.3908170000 8.7999990000

C53 27.0193340000 53.4836890000 9.2222590000

N54 27.0357450000 53.8206000000 10.6609950000

C55 26.3114320000 53.2341160000 11.6219020000

N56 25.4632570000 52.2341490000 11.3556880000

N57 26.3946340000 53.6745250000 12.8945130000

C58 24.1709720000 65.7240050000 15.0359970000

C59 25.4811840000 65.0159260000 14.6413430000

C60 25.3074840000 63.5566680000 14.2654380000

C61 25.6542330000 62.5291430000 15.1522230000

C62 24.7922840000 63.1862740000 13.0122540000

C63 25.5045370000 61.1802580000 14.8118960000

C64 24.6347780000 61.8482620000 12.6545150000

C65 24.9967460000 60.8426510000 13.5546810000

O66 24.7603670000 59.5242620000 13.1774810000

C67 19.2469760000 58.8630060000 17.5249980000

C68 20.6821280000 59.2865210000 17.8717900000

C69 21.6426980000 58.8772470000 16.8046870000

N70 22.0064380000 57.5549570000 16.5848560000

C71 22.2559840000 59.5994640000 15.8172120000

C72 22.8051680000 57.4815960000 15.5148460000

N73 22.9638070000 58.7126750000 15.0313650000

H74 25.0054600000 51.7649270000 12.1356310000

H75 25.2608310000 51.9422240000 10.4120870000

H76 27.2479710000 54.1136120000 13.2337770000

H77 25.8846130000 53.1508440000 13.6046990000

H78 27.5843920000 54.6222250000 10.9422030000

H79 29.7394870000 52.9697030000 9.9766080000

H80 29.6410840000 53.7515290000 8.4014860000

H81 27.7860350000 52.1502110000 7.7525800000

H82 27.8213850000 51.4734780000 9.3742010000

H83 27.2389170000 54.4166570000 8.6947180000

H84 25.9929670000 53.2200790000 8.9418240000

H85 31.4845900000 52.0578970000 8.4445680000

H86 30.2502640000 51.5902470000 7.2672630000

H87 30.3291690000 50.7768000000 8.8385970000

H88 18.9223910000 59.3150090000 16.5826990000

H89 18.5546240000 59.1819390000 18.3102450000

H90 19.1630790000 57.7748100000 17.4270600000

H91 20.7385570000 60.3735930000 17.9875040000

H92 20.9854000000 58.8537490000 18.8338940000

H93 21.7042630000 56.7312240000 17.1265330000

H94 23.2791520000 56.5878140000 15.1431360000

H95 27.1687150000 57.3081330000 14.1153490000

H96 24.3621750000 66.7717630000 15.2898060000

H97 23.7090590000 65.2427200000 15.9055990000

H98 23.4439380000 65.7037640000 14.2163770000

H99 25.9370400000 65.5526150000 13.7995670000

H100 26.1924700000 65.0903490000 15.4731890000

H101 26.0431810000 62.7830060000 16.1353820000

H102 24.5207920000 63.9586540000 12.2965800000

H103 24.2442280000 61.5693600000 11.6806620000

H104 25.7924760000 60.4045080000 15.5173500000

H105 32.7393130000 55.7972250000 23.1728660000

H106 31.0261860000 55.5656940000 22.8237720000

H107 31.8992450000 56.9345030000 22.1113870000

H108 33.3754830000 55.2951830000 20.8437520000

H109 32.4255970000 53.9617530000 21.4748730000

H110 31.3966800000 56.1969530000 19.6514360000

H111 31.8163810000 54.5660070000 19.1329490000

H112 30.0199250000 53.6429760000 20.6693450000

H113 29.5235890000 55.2822780000 21.0504860000

H114 29.4583980000 54.0037380000 18.2579880000

H115 28.0758700000 54.2301730000 19.3277020000

H116 28.5409010000 56.6292890000 19.1937250000

H117 29.4065890000 62.3412310000 17.7626720000

H118 28.3472820000 62.3540630000 16.3432450000

H119 29.9022960000 61.5297080000 16.2836330000

H120 26.9810000000 60.8208820000 20.5344380000

H121 28.5600600000 61.4414230000 20.1327830000

H122 26.8639280000 63.2607180000 20.2422300000

H123 25.9236810000 62.4980190000 18.9514520000

H124 27.5261050000 63.1645550000 18.6107960000

H125 31.0268630000 59.2420780000 13.5532510000

H126 31.8250080000 57.9304970000 12.7199230000

H127 29.0226890000 59.0037640000 12.0108360000

H128 29.8593160000 57.6934170000 11.1571360000

H129 30.5135050000 59.3378250000 11.1137330000

H130 29.6457140000 59.6990920000 15.1141350000

H131 25.9163730000 59.0567460000 20.2584180000

H132 29.4542640000 53.3629710000 14.3010360000

H133 29.2443690000 50.9871910000 15.2205970000

H134 27.9716520000 51.1731140000 14.0168620000

H135 27.7409710000 50.0841950000 15.3892680000

H136 26.2804460000 50.0155540000 17.1092930000

H137 25.4687700000 51.0160880000 18.2947980000

H138 23.7950820000 50.0227910000 16.7844250000

H139 23.8354580000 51.7904190000 16.5164150000

H140 24.6860330000 53.0621920000 18.5223340000

H141 31.4943700000 55.7379410000 12.0863540000

H142 30.0774880000 54.6947970000 11.9566220000

H143 31.3649630000 54.2939630000 13.0912390000

H144 24.7031030000 57.9096430000 21.9988960000

H145 23.3405660000 56.7922040000 22.0023400000

H146 23.2866770000 58.2529730000 21.0108060000

H147 23.4523990000 53.4412770000 20.1899650000

H148 23.2616890000 54.5724790000 21.5026330000

H149 21.0888370000 54.2324780000 20.7111040000

H150 21.5245750000 55.8370860000 20.0916300000

H151 25.2352240000 49.2536840000 13.7822500000

H152 20.9263650000 52.7982350000 17.6093780000

N153 26.1490780000 57.3542430000 14.0863980000

O154 25.9382250000 56.6956190000 15.8108040000

H155 29.6670170000 56.3449580000 18.0547260000

H156 22.2491900000 60.6580040000 15.6111800000

H157 25.8477810000 56.4796670000 13.6499990000

H158 23.5687980000 58.9791430000 14.2170640000

H159 25.6415050000 57.5581730000 16.1501870000

H160 25.4273690000 58.8750910000 13.5560600000

The NH_2_^.^ product fter the cleavage of the second N-O bond.

Energies: E= -3399.037156, solv = -0.141791, disp = -183.72 Z_0_ = xx

Fe1 27.3200819032 56.3153735958 16.9524683964

C2 25.2906414497 53.8367666265 18.1408597434

C3 28.9059083435 54.0794675707 14.9331048330

C4 29.0906136530 58.8905985967 15.5537783442

C5 26.2006418425 58.4044202106 19.4080910199

C6 26.2778578254 53.4838504540 17.2269476336

C7 26.5268649607 52.1229612552 16.7933532096

C8 27.5515381349 52.1757955904 15.8841880599

C9 27.9306081909 53.5799250256 15.7909588167

C10 28.1569782339# 51.0510111305# 15.0929935526#

C11 25.7085698904 50.9474593166 17.2327616035

C12 24.3883181841 50.8633646287 16.4211165242

C13 24.7229795510# 50.6400113114# 14.9749948153#

O14 25.0762911871 49.3636410731 14.7548736430

O15 24.7071136136 51.4750083339 14.0794439696

C16 29.2462254901 55.4212766540 14.7823622614

C17 30.1135258747 55.9378749328 13.7391203587

C18 30.1420235226 57.3040985925 13.8968128433

C19 29.3001439195 57.6116067153 15.0401955668

C20 30.7959743521# 55.1300164736# 12.6669937906#

C21 30.8348531848 58.2993425417 13.0113293306

C22 30.0089716802# 58.6030164631# 11.7499928684#

C23 28.3478999494 59.1962163426 16.6937551710

C24 28.3554858694 60.4771175124 17.3832474597

C25 27.6478828615 60.2905429706 18.5483023615

C26 27.1182299376 58.9353342572 18.4998722403

C27 29.0409715257# 61.7380137041# 16.9269939800#

C28 27.5254553651 61.2510678251 19.7019694723

C29 26.9359740211# 62.6240158745# 19.3539940134#

C30 25.5976856891 57.1479106595 19.3343715760

C31 24.5312651142 56.6802228251 20.2102478287

C32 24.2164531681 55.4049777184 19.7964661411

C33 25.1339514305 55.0936087174 18.7131265870

C34 23.9419772726# 57.4440117376# 21.3619974448#

C35 23.2225619263 54.4532522359 20.4173985610

C36 21.7193899730 54.6548042074 20.0491739618

C37 21.3749769009# 54.3240098410# 18.6159944137#

O38 21.1403120461 53.0200384170 18.4176423344

O39 21.3326355003 55.1343673807 17.6978887997

N40 27.1516544018 54.3435211348 16.6227014256

N41 28.7654033648 56.4521800604 15.5589892572

N42 27.5850191735 58.2817453134 17.3816973229

N43 25.9376610665 56.1682563127 18.4351739581

C44 31.9819766750# 55.8750149992# 22.3859916376#

C45 32.3129315474 54.9840093296 21.1819435703

C46 31.3498630709 55.1356631196 19.9911003469

C47 29.9028954963 54.7238801358 20.3028884190

C48 28.9691406643 54.6811901436 19.0880502301

N49 28.7811154551 55.9951632162 18.4195369318

C50 30.4419720987# 51.7440156295# 8.3349938659#

C51 29.4014527562 52.7653059196 8.8182507157

C52 27.9662734080 52.2209024931 8.7723152358

C53 26.8701472718 53.2441734504 9.1125796508

N54 26.8512219582 53.7129679278 10.5117048057

C55 26.1334145988 53.1522392083 11.5031668952

N56 25.5959206696 51.9282382422 11.3368930219

N57 25.9363452302 53.8126232105 12.6408293457

C58 24.1709719879# 65.7240051595# 15.0359966333#

C59 25.1331566726 65.2770972455 13.9197489411

C60 25.1003544195 63.7801678463 13.6962413116

C61 25.7903210567 62.9123694678 14.5576235837

C62 24.3468053229 63.2121926356 12.6610136714

C63 25.7293191492 61.5270569235 14.4073765501

C64 24.2666411531 61.8276737048 12.4945801159

C65 24.9563366670 60.9974366993 13.3747685727

O66 24.8697138954 59.6005097866 13.2840164926

C67 19.2469767004# 58.8630056755# 17.5249979834#

C68 20.6104375147 59.3983000253 17.9947728223

C69 21.7526962012 59.0037040398 17.1097603684

N70 22.3018961740 57.7236393639 17.0807499066

C71 22.4710286546 59.7320160757 16.2017416522

C72 23.3145769496 57.6770574607 16.2096579722

N73 23.4279749910 58.8898545888 15.6628877093

H74 25.1215483969 51.5122054638 12.1352863341

H75 25.9466254058 51.3164558815 10.6151835400

H76 26.1573751864 54.8227609931 12.7286781743

H77 25.5001216783 53.3327765046 13.4214649338

H78 27.2295233975 54.6293410762 10.7122749734

H79 29.6428525463 53.0824752242 9.8427229225

H80 29.4594299867 53.6671557554 8.1923588474

H81 27.7501860450 51.8550780196 7.7595349822

H82 27.8851953402 51.3441390773 9.4327816901

H83 26.9939433274 54.1368883648 8.4920738149

H84 25.8786797726 52.8395192973 8.8793952231

H85 31.4521654534 52.1624444402 8.3815231761

H86 30.2507452307 51.4486618039 7.2973423848

H87 30.4271030834 50.8369541728 8.9506011887

H88 19.0027773314 59.2350370968 16.5252921641

H89 18.4594873686 59.1878403984 18.2111798082

H90 19.2352627608 57.7678466859 17.4933413150

H91 20.5849017379 60.4922336479 18.0367133970

H92 20.8138717322 59.0581865967 19.0185385455

H93 21.9437947166 56.8823102741 17.5551181404

H94 24.0098899805 56.8614923935 16.0244697175

H95 27.5196599395 56.7877147593 13.3722637210

H96 24.2194219519 66.8086020052 15.1773775344

H97 24.4243963051 65.2473690957 15.9898498113

H98 23.1357989932 65.4610210208 14.7918231525

H99 24.8764901630 65.7930268559 12.9873975819

H100 26.1537395747 65.5883889681 14.1751607228

H101 26.3896076642 63.3295957130 15.3629357098

H102 23.8085560381 63.8570079970 11.9717510693

H103 23.6723878053 61.4081581493 11.6848808908

H104 26.2808099590 60.8675673871 15.0716370506

H105 32.7412882870 55.7688888213 23.1675615817

H106 31.0157319814 55.6186926282 22.8351125198

H107 31.9495549745 56.9335492699 22.0980629860

H108 33.3291521637 55.2109764865 20.8359674754

H109 32.3276828253 53.9312606921 21.4966785238

H110 31.3773218922 56.1808973268 19.6462295235

H111 31.7199501163 54.5241433848 19.1549441229

H112 29.9023111223 53.7201873291 20.7509441724

H113 29.4715944129 55.3923300886 21.0613481086

H114 29.3489668198 53.9884129766 18.3312644068

H115 27.9781319533 54.3296637323 19.3851207020

H116 28.5614353828 56.7025828338 19.1228430855

H117 29.4670576417 62.2851204062 17.7731628777

H118 28.3470018571 62.4129036876 16.4121930782

H119 29.8608105613 61.5271300913 16.2337898036

H120 26.9319254141 60.8004383375 20.5043857818

H121 28.5268932102 61.3966139780 20.1329558274

H122 26.8553094662 63.2406693712 20.2552187202

H123 25.9317174111 62.5246247109 18.9259324714

H124 27.5536423707 63.1698976541 18.6375138131

H125 31.0333956407 59.2295235840 13.5544317727

H126 31.8179847732 57.9124851229 12.7181717617

H127 29.0309826583 59.0220646014 12.0145262003

H128 29.8397133740 57.6935808171 11.1630759325

H129 30.5214600603 59.3267909034 11.1072559160

H130 29.6080372879 59.7078612350 15.0660886621

H131 25.8840944043 59.0482864104 20.2203843424

H132 29.4143420098 53.3676541693 14.2916137754

H133 29.2445993998 51.0023529516 15.2222151847

H134 27.9668784366 51.1737763449 14.0182277499

H135 27.7483850991 50.0831887799 15.3949897798

H136 26.2628159038 50.0121979226 17.1104905081

H137 25.4575875265 51.0184825496 18.2959529864

H138 23.7770710494 50.0280351026 16.7772093817

H139 23.8232919531 51.7937344055 16.5086570475

H140 24.6404854320 53.0468216336 18.4965361099

H141 31.4700710516 55.7522685766 12.0734375764

H142 30.0712865455 54.6736772141 11.9790634203

H143 31.3915944529 54.3133696285 13.0924851992

H144 24.7229105526 57.8393425995 22.0220097710

H145 23.2927031917 56.8082165662 21.9705419850

H146 23.3435557124 58.3014165123 21.0264606753

H147 23.4962420266 53.4159887461 20.2003526089

H148 23.2768346668 54.5464084180 21.5081076518

H149 21.1193340942 54.0161581310 20.7045592530

H150 21.4315785644 55.6947660112 20.2246046217

H151 25.3057846488 49.2518148388 13.8136899299

H152 20.9077988369 52.9022835749 17.4756124275

N153 26.6168145623 56.5997960267 12.9229327152

O154 26.0230053392 56.6910767476 15.6915769539

H155 29.6535096677 56.2983129817 17.9852068628

H156 22.3887534487 60.7660139915 15.9070885386

H157 26.1400693743 57.5118035222 12.9237865063

H158 24.0764039762 59.1200634597 14.9011491370

H159 24.3058583949 59.3797990859 12.5232050699

H160 26.2491266814 57.5974812769 15.4221571281

The NH_3_ product after the cleavage of the second N-O bond.

Energies: E= -3399.075282, solv = -0.136396, disp = -184.49 Z_0_ = xx

Fe1 27.2608288133 56.2996274483 16.9081390570

C2 25.2939072255 53.8106064421 18.1676717382

C3 28.9055794810 54.0720272009 14.9511584950

C4 29.0354049817 58.8901689067 15.5067542921

C5 26.1235365499 58.4109057796 19.3419477832

C6 26.3029766530 53.4547967886 17.2768403162

C7 26.5475844027 52.0984472132 16.8252034828

C8 27.5741434708 52.1613970809 15.9169922553

C9 27.9595229978 53.5625065806 15.8394830338

C10 28.1569781644# 51.0510111634# 15.0929935523#

C11 25.7316476124 50.9107560243 17.2397440724

C12 24.4076992840 50.8173440160 16.4322029878

C13 24.7229797688# 50.6400113026# 14.9749946272#

O14 25.0058451007 49.3633397644 14.6813752159

O15 24.7499757542 51.5249331033 14.1272545841

C16 29.2062839509 55.4187042207 14.7589441031

C17 30.0860526841 55.9332654897 13.7220932128

C18 30.1154459396 57.2957419403 13.8815080994

C19 29.2454630654 57.6064034445 15.0029194250

C20 30.7959743487# 55.1300164740# 12.6669937803#

C21 30.8269962178 58.2875173680 13.0101636716

C22 30.0089716929# 58.6030164454# 11.7499929164#

C23 28.2783709540 59.2036184584 16.6345982723

C24 28.3107324294 60.4785974016 17.3405378404

C25 27.5886552802 60.2881579996 18.4966482456

C26 27.0354739767 58.9420201576 18.4280531872

C27 29.0409715315# 61.7380136802# 16.9269939837#

C28 27.4878427188 61.2280297443 19.6698888907

C29 26.9359740165# 62.6240158842# 19.3539940048#

C30 25.5331161222 57.1474336918 19.2864752826

C31 24.4935605717 56.6740616546 20.1937478105

C32 24.1883628345 55.3906647489 19.7990422979

C33 25.0985023163 55.0787459126 18.7047942570

C34 23.9419772690# 57.4440117244# 21.3619974279#

C35 23.2107147422 54.4314129028 20.4332562534

C36 21.7039278334 54.6053029853 20.0649696768

C37 21.3749769911# 54.3240098710# 18.6159945666#

O38 21.0249602842 53.0486958146 18.3929004010

O39 21.4381385949 55.1470747835 17.7125392203

N40 27.1904919784 54.3129337506 16.6901578221

N41 28.7010109352 56.4525092429 15.5115353685

N42 27.4951329247 58.2935078465 17.3048790592

N43 25.8692224809 56.1660314532 18.3904635014

C44 31.9819766880# 55.8750149870# 22.3859916473#

C45 32.2973552879 55.0124141467 21.1587278729

C46 31.3253400389 55.2057468571 19.9823306056

C47 29.8690271345 54.8536189085 20.3209958205

C48 28.9261055399 54.7855322792 19.1162052581

N49 28.7494497885 56.0722610171 18.3946699278

C50 30.4419720003# 51.7440156076# 8.3349939394#

C51 29.6797380226 52.8739674389 9.0506832436

C52 28.1642148701 52.6194253272 9.1227897830

C53 27.2934276444 53.8028979908 9.5947808337

N54 27.2400842212 54.0708699672 11.0508875647

C55 26.3529358067 53.5112939151 11.8912002873

N56 25.6890650460 52.4028741986 11.5092781459

N57 26.0854283659 54.0495403186 13.0806626044

C58 24.1709720720# 65.7240051789# 15.0359966067#

C59 24.8954289436 65.3742289739 13.7125975339

C60 24.8491132843 63.8976090257 13.4357073138

C61 25.6688397850 63.0114707797 14.1887172426

C62 23.9412892978 63.3487921824 12.4946545307

C63 25.5489062386 61.6504494635 14.0701630745

C64 23.8072254107 61.9886470018 12.3438937891

C65 24.5722908096 61.0748850217 13.1664784743

O66 24.3569701170 59.8242158510 13.1177598627

C67 19.2469764570# 58.8630056805# 17.5249979471#

C68 20.6362313028 59.2662619546 18.0468113259

C69 21.7593116900 58.9402823064 17.1082519854

N70 22.4649308327 57.7414872741 17.1426850991

C71 22.3255437256 59.6659452093 16.0966398085

C72 23.4246102724 57.7378047009 16.2102871318

N73 23.3466858250 58.9010752174 15.5622103505

H74 25.1280639691 51.9251750168 12.2131036825

H75 26.0873866628 51.8251421583 10.7823515152

H76 26.3783694841 54.9874730778 13.3563381975

H77 25.5753254674 53.5032808971 13.7697266910

H78 27.7749361174 54.8443461628 11.4203685723

H79 30.0848779617 53.0116724131 10.0624974358

H80 29.8541544220 53.8182864183 8.5159142553

H81 27.8064012255 52.3665561270 8.1157311774

H82 27.9695153168 51.7300250673 9.7410349057

H83 27.6510059069 54.7260944200 9.1316770422

H84 26.2604887335 53.6639297528 9.2605170030

H85 31.5141745737 51.9592610231 8.3043545921

H86 30.0928312987 51.6278347876 7.3034311495

H87 30.3074683514 50.7842334768 8.8473490955

H88 19.0158811402 59.3769653814 16.5869579042

H89 18.4778952319 59.1282048609 18.2565067980

H90 19.1893144214 57.7842344679 17.3446529576

H91 20.6580891280 60.3442690519 18.2409545697

H92 20.8291073585 58.7804164600 19.0112181755

H93 22.2215619070 56.9098681268 17.6913850071

H94 24.2083746336 56.9907688094 16.0418466083

H95 27.3525067914 58.1581957072 13.1079411044

H96 24.2309346541 66.7997863867 15.2234474935

H97 24.6260642368 65.2069539508 15.8856835907

H98 23.1143823666 65.4450118743 14.9901875313

H99 24.4356978658 65.9261391175 12.8875274479

H100 25.9394999937 65.7039909386 13.7810332765

H101 26.3900331729 63.4354668982 14.8815525065

H102 23.3343387375 64.0236152108 11.8992567818

H103 23.1021348464 61.5551762096 11.6430898854

H104 26.1626632626 60.9732933347 14.6559410400

H105 32.7472314324 55.7469939973 23.1584630567

H106 31.0187271351 55.6122899667 22.8366045508

H107 31.9522804606 56.9405318808 22.1249588043

H108 33.3141577592 55.2353545346 20.8118950764

H109 32.2998969735 53.9522264445 21.4473153627

H110 31.3889016676 56.2488614899 19.6365335610

H111 31.6572625737 54.5795590745 19.1407734956

H112 29.8412245463 53.8696105537 20.8104555570

H113 29.4653728976 55.5657994507 21.0542688659

H114 29.2871550816 54.0544833226 18.3871831283

H115 27.9316468710 54.4631883741 19.4343301945

H116 28.5297203309 56.8071558264 19.0686668759

H117 29.5062708704 62.2242083686 17.7899239842

H118 28.3624724181 62.4656453204 16.4649399507

H119 29.8366452369 61.5286542905 16.2057242275

H120 26.8813057954 60.7764994476 20.4617530514

H121 28.4919745636 61.3377761713 20.1055496781

H122 26.8711528378 63.2213869501 20.2693663915

H123 25.9296100423 62.5610590812 18.9238585957

H124 27.5688381502 63.1696637105 18.6504775637

H125 31.0337836565 59.2133486826 13.5577200828

H126 31.8066451123 57.8923819862 12.7174810342

H127 29.0479135704 59.0641968358 12.0044209643

H128 29.8063248157 57.6917544459 11.1760017639

H129 30.5433145895 59.2984849453 11.0934562350

H130 29.5828421853 59.6989830508 15.0368759239

H131 25.8141968893 59.0549076856 20.1569210911

H132 29.4197694834 53.3578647630 14.3162320879

H133 29.2453191654 50.9837301552 15.2086758213

H134 27.9570342458 51.2010149960 14.0235103464

H135 27.7399872943 50.0803026176 15.3743410421

H136 26.2957503770 49.9826723253 17.1054050503

H137 25.4776000861 50.9619915586 18.3036051042

H138 23.8170824186 49.9632384693 16.7770597225

H139 23.8265584273 51.7346546828 16.5494043793

H140 24.6545677932 53.0142905840 18.5293349616

H141 31.3968100499 55.7720709616 12.0184589893

H142 30.0976256231 54.5721494455 12.0284311112

H143 31.4728742459 54.3871450715 13.1077481686

H144 24.7455473262 57.8059644357 22.0141044845

H145 23.2822064051 56.8213148276 21.9728910160

H146 23.3660527751 58.3229988421 21.0442334585

H147 23.5002028012 53.3968332166 20.2233562356

H148 23.2642121120 54.5334930672 21.5233084670

H149 21.1167663720 53.9328978240 20.6968527253

H150 21.3929513203 55.6328927930 20.2750353337

H151 25.2209272123 49.2947970253 13.7321932274

H152 20.8139584434 52.9653091898 17.4421764854

N153 26.5115620896 57.6141135174 12.8718752706

O154 25.9598368130 56.4156060125 15.6208063243

H155 29.6231096389 56.3536039070 17.9486762682

H156 22.0899806837 60.6495158689 15.7233428852

H157 26.6126332328 57.3628110148 11.8862108622

H158 23.9227505114 59.1529664916 14.7463592246

H159 26.2952452747 56.8657476383 14.8225992178

H160 25.7367953029 58.2737189315 12.9144271757

The Fe-OH tyrosine product.

Energies: E= -3343.153768, solv = -0.142635, disp = -184.79 Z_0_ = 850.13

Fe1 27.3230404968 56.3405596593 16.9678421572

C2 25.3074537028 53.8702944001 18.1316991840

C3 28.9027242305 54.0798970754 14.8991617253

C4 29.0671783319 58.8796471402 15.5644333901

C5 26.2530533478 58.4119650490 19.4752084574

C6 26.2869283145 53.5067436673 17.2143008924

C7 26.5273366883 52.1438497829 16.7875409164

C8 27.5552157904 52.1844144828 15.8772481844

C9 27.9403640030 53.5816511347 15.7765729519

C10 28.1569781034# 51.0510113221# 15.0929936245#

C11 25.7078129528 50.9706351530 17.2316249285

C12 24.3882748196 50.8751062401 16.4182173279

C13 24.7229798051# 50.6400113339# 14.9749945517#

O14 25.0062872449 49.3512927528 14.7437063869

O15 24.7639700438 51.4846826728 14.0846592155

C16 29.2210530449 55.4279988982 14.7457265610

C17 30.1033663696 55.9505935166 13.7209792134

C18 30.1282380962 57.3124383781 13.8901599935

C19 29.2624303697 57.6086139760 15.0237760701

C20 30.7959742010# 55.1300165046# 12.6669939131#

C21 30.8266400650 58.3133341620 13.0189522368

C22 30.0089716797# 58.6030164592# 11.7499928837#

C23 28.3610326742 59.1839217771 16.7320189861

C24 28.3904105615 60.4716589361 17.4162497951

C25 27.7042067934 60.2955427965 18.5957007201

C26 27.1714617344 58.9399383584 18.5666148102

C27 29.0409715063# 61.7380136405# 16.9269939849#

C28 27.5852591005 61.2830039742 19.7271094214

C29 26.9359740207# 62.6240158689# 19.3539940031#

C30 25.6364096113 57.1625626377 19.3759765754

C31 24.5593956836 56.6870939884 20.2310012095

C32 24.2466770489 55.4184440107 19.8050776926

C33 25.1652060681 55.1221354776 18.7187471783

C34 23.9419772961# 57.4440116877# 21.3619973707#

C35 23.2354682574 54.4750276604 20.4095064329

C36 21.7437354036 54.7161134658 20.0275234185

C37 21.3749771005# 54.3240100331# 18.6159944682#

O38 21.3406335024 52.9931918845 18.4370716909

O39 21.1360257933 55.1009767089 17.7014350791

N40 27.1667674865 54.3598346841 16.6027409122

N41 28.7192313257 56.4470969910 15.5198404872

N42 27.6167313281 58.2779778070 17.4461392423

N43 25.9734706668 56.1984541154 18.4609569528

C44 31.9819766543# 55.8750149872# 22.3859916118#

C45 32.3860239533 55.0486711519 21.1580987250

C46 31.4393541172 55.1922458053 19.9530673850

C47 30.0138875250 54.6859323758 20.2193093938

C48 29.0944698788 54.6630661293 18.9929646025

N49 28.8351205768 56.0032268176 18.4034225322

C50 30.4419719781# 51.7440156015# 8.3349939863#

C51 29.4306741259 52.7737150738 8.8550889461

C52 27.9748087973 52.3037305014 8.7186157771

C53 26.9337895671 53.3615142761 9.1102649774

N54 26.9405190768 53.7301632440 10.5401427327

C55 26.2625579232 53.1280389886 11.5248326605

N56 25.4158514713 52.1171248790 11.2872411604

N57 26.4026151314 53.5503111259 12.7942028783

C58 24.1709721130# 65.7240049742# 15.0359966122#

C59 25.4873039235 65.1864473091 14.4445933500

C60 25.5915899210 63.6723028446 14.4292469999

C61 25.5684759342 62.9367195697 15.6252673277

C62 25.7257246773 62.9570241559 13.2305819924

C63 25.6655775690 61.5438151577 15.6284041392

C64 25.8388138803 61.5633536518 13.2163775320

C65 25.8126734935 60.8583399228 14.4180713174

O66 25.8384577618 59.4610824999 14.3661273274

C67 19.2469765418# 58.8630055864# 17.5249979895#

C68 20.5634485786 59.6061393411 17.7857650726

C69 21.7399451310 59.0492568393 17.0453844890

N70 22.1146446354 57.7084893842 17.0883921218

C71 22.6852533130 59.6683582017 16.2741079489

C72 23.2407519295 57.5309184304 16.3879795105

N73 23.5979469755 58.7070241655 15.8825010914

H74 24.9586563469 51.6754818279 12.0824681132

H75 25.1195327544 51.8784989412 10.3533622800

H76 27.2409126543 54.0359392408 13.0983042366

H77 25.8914796413 53.0561232621 13.5237586491

H78 27.4823838552 54.5420742040 10.8046553059

H79 29.6485066597 52.9995367387 9.9092690494

H80 29.5552856589 53.7164421501 8.3033308335

H81 27.7758850227 52.0337524182 7.6730378991

H82 27.8132298619 51.3909611712 9.3082751090

H83 27.1104033270 54.2913550236 8.5616456785

H84 25.9212729594 53.0449869843 8.8357888224

H85 31.4672028255 52.1079647878 8.4535426991

H86 30.2823817378 51.5386584785 7.2707093279

H87 30.3591760802 50.7943100499 8.8762369308

H88 18.9759763269 58.8962353233 16.4651580073

H89 18.4365397935 59.3241197148 18.0964871956

H90 19.3050914283 57.8110200113 17.8276520544

H91 20.4626828243 60.6591723549 17.5032187371

H92 20.7823259072 59.5999923939 18.8628459979

H93 21.6040726542 56.9236856302 17.5093495454

H94 23.8204993701 56.6274641985 16.2770772533

H95 24.1679882332 66.8184922749 15.0273658506

H96 24.0336808936 65.3989552991 16.0734465857

H97 23.3081125839 65.3777258633 14.4568173083

H98 25.6069587146 65.5638521698 13.4225795356

H99 26.3281431240 65.5976082652 15.0202718795

H100 25.4728662724 63.4588584659 16.5741122559

H101 25.7539176365 63.4978923446 12.2882734250

H102 25.9443094855 61.0207655391 12.2822164666

H103 25.6473068527 60.9952407746 16.5655701690

H104 32.7310463600 55.7825003777 23.1789825192

H105 31.0233936546 55.5490787642 22.8052271731

H106 31.8957157353 56.9399371331 22.1352011024

H107 33.3946058779 55.3438597469 20.8427334792

H108 32.4530528062 53.9873596561 21.4349526358

H109 31.4120266249 56.2504996580 19.6507349535

H110 31.8605781373 54.6375166095 19.1015233677

H111 30.0629165828 53.6601438971 20.6110916532

H112 29.5383529404 55.2862988247 21.0076083373

H113 29.5166306426 54.0377856335 18.2003670252

H114 28.1233904291 54.2393167953 19.2614202449

H115 28.5940891806 56.6555271790 19.1511058544

H116 29.4657753628 62.3096181265 17.7578314385

H117 28.3178346771 62.3776213989 16.4088140755

H118 29.8572349450 61.5341790517 16.2266261318

H119 27.0341843820 60.8365282879 20.5619180878

H120 28.5941706612 61.4772425280 20.1187520980

H121 26.8944521306 63.2808323548 20.2291501531

H122 25.9092488445 62.4772420516 18.9984931748

H123 27.4899895376 63.1487919052 18.5731509979

H124 31.0088430729 59.2457356614 13.5637199118

H125 31.8167906349 57.9344724234 12.7384182460

H126 29.0203468564 59.0022214203 12.0035104169

H127 29.8641111628 57.6905225239 11.1608761507

H128 30.5162959087 59.3359650502 11.1131727586

H129 29.5795013565 59.7012867025 15.0784577541

H130 25.9432269199 59.0496605623 20.2951795986

H131 29.4397367493 53.3653505187 14.2831005134

H132 29.2382183983 50.9727097115 15.2570768645

H133 28.0052448535 51.1822705770 14.0134200819

H134 27.7155163673 50.0913370052 15.3742561099

H135 26.2639408371 50.0349925043 17.1198238562

H136 25.4514894247 51.0492529920 18.2927036434

H137 23.7795619754 50.0420646006 16.7827031902

H138 23.8227880394 51.8058991880 16.4988492208

H139 24.6496825627 53.0863143584 18.4867020098

H140 31.5310820136 55.7274196283 12.1219070560

H141 30.0910130587 54.7269825734 11.9254510902

H142 31.3275160553 54.2751766854 13.1011522483

H143 24.7023409691 57.8896605371 22.0140268010

H144 23.3210801203 56.7949655767 21.9861171791

H145 23.3051679257 58.2663810571 21.0091081138

H146 23.4901254554 53.4345571620 20.1861621410

H147 23.2860721905 54.5599213438 21.5009545349

H148 21.1196176978 54.1257230147 20.7074606518

H149 21.4856794954 55.7696807304 20.1573133933

H150 25.2459843447 49.2381036773 13.8044612620

H151 21.0499116356 52.8322347407 17.5180744603

O152 26.0133947808 56.8016638768 15.7631363068

H153 29.6875926253 56.3689684445 17.9782884518

H154 22.7896207926 60.7017245659 15.9848253587

H155 24.4531193199 58.8548756887 15.3172625409

H156 26.4916445607 59.0908850675 14.9913759504

H157 26.2380703550 56.3324888356 14.9426731993

The Fe-H_2_O tyrosine product.

Energies: E= -3343.753190, solv = -0.145565, disp = -185.06 Z_0_ = 856.79

Fe1 27.3654749082 56.3150322017 17.0138244449

C2 25.2896405557 53.8494660262 18.1311066457

C3 28.8953282273 54.0835567524 14.9050976973

C4 29.0900614918 58.8823527498 15.5669682638

C5 26.1939251964 58.4113950983 19.4147881143

C6 26.2713665995 53.4884395996 17.2126724908

C7 26.5263782137 52.1248110817 16.7917149891

C8 27.5533318672 52.1749173608 15.8825023123

C9 27.9274941314 53.5772411411 15.7793563266

C10 28.1569781238# 51.0510112524# 15.0929935879#

C11 25.7164988623 50.9444906230 17.2326677648

C12 24.3940469344 50.8470769995 16.4234391508

C13 24.7229798277# 50.6400113321# 14.9749946023#

O14 25.0487416604 49.3651399879 14.7250076401

O15 24.7230840356 51.4940746117 14.0921257169

C16 29.2238369841 55.4331011700 14.7489809771

C17 30.1096769659 55.9510323130 13.7202198985

C18 30.1479986614 57.3106826167 13.8892913338

C19 29.2861456965 57.6114176458 15.0264673795

C20 30.7959742512# 55.1300164787# 12.6669938627#

C21 30.8401144177 58.3089612004 13.0092541435

C22 30.0089716811# 58.6030164363# 11.7499929178#

C23 28.3507333699 59.1900013374 16.7130237080

C24 28.3688296488 60.4769083974 17.4011050641

C25 27.6593522584 60.2971746584 18.5647797200

C26 27.1197854125 58.9426267017 18.5149894257

C27 29.0409714844# 61.7380135809# 16.9269939866#

C28 27.5400541692 61.2622505442 19.7138188789

C29 26.9359740295# 62.6240158420# 19.3539939965#

C30 25.5824338818 57.1564065181 19.3315989396

C31 24.5219178458 56.6823620810 20.2113125063

C32 24.2112642196 55.4085157434 19.7992742949

C33 25.1257878623 55.1078102013 18.7053629057

C34 23.9419773121# 57.4440116871# 21.3619973845#

C35 23.2177444382 54.4585785986 20.4200112389

C36 21.7175693602 54.6693414936 20.0441324728

C37 21.3749771327# 54.3240099888# 18.6159945280#

O38 21.2248886513 53.0074401613 18.4172925379

O39 21.2553370742 55.1269153001 17.6963761286

N40 27.1393377614 54.3520983433 16.5937159122

N41 28.7284414667 56.4546391978 15.5231781119

N42 27.5797995262 58.2830670074 17.3982069998

N43 25.9177489473 56.1895217330 18.4194141456

C44 31.9819766712# 55.8750149867# 22.3859916269#

C45 32.3525060870 55.0110311784 21.1736992610

C46 31.3976123378 55.1525321015 19.9750451324

C47 29.9651345807 54.6737230944 20.2565380103

C48 29.0386952100 54.6616062896 19.0346442054

N49 28.7965417572 56.0004855156 18.4389869264

C50 30.4419719791# 51.7440156069# 8.3349939712#

C51 29.4872362320 52.7881422984 8.9264632813

C52 28.0096399728 52.3973236698 8.7772219995

C53 27.0260936386 53.4803080066 9.2409138466

N54 27.0579365511 53.7589304905 10.6901431126

C55 26.2921870633 53.1925398859 11.6301086012

N56 25.3613206362 52.2759123681 11.3318733940

N57 26.4040541898 53.5850513623 12.9162935419

C58 24.1709720829# 65.7240051801# 15.0359966125#

C59 25.3251997966 65.1946314574 14.1687980442

C60 25.3713499169 63.6825371732 14.1085226494

C61 25.6813789157 62.9330841900 15.2550147661

C62 25.0954416010 62.9839503416 12.9269226214

C63 25.7193219085 61.5391619794 15.2331656850

C64 25.1250191712 61.5872175972 12.8818812931

C65 25.4393103076 60.8783600584 14.0377624858

O66 25.4588299044 59.4756189322 14.0587821428

C67 19.2469764244# 58.8630056274# 17.5249979232#

C68 20.5247914380 59.4214277270 18.1706872528

C69 21.7537887514 59.0357869865 17.4125056500

N70 22.1859638071 57.7188445764 17.3008838809

C71 22.6327361020 59.7767753006 16.6710487935

C72 23.2767026671 57.6613190130 16.5362699041

N73 23.5649797756 58.9009206819 16.1429346199

H74 24.9090294480 51.7856966072 12.1014920916

H75 25.2363424643 51.9310730405 10.3928091911

H76 27.2856488299 53.9649385560 13.2621389772

H77 25.8872303809 53.0409771842 13.6067390585

H78 27.7004525357 54.4725502634 11.0087575042

H79 29.7251942945 52.9396592630 9.9893534757

H80 29.6521299831 53.7550367684 8.4298372277

H81 27.7903520834 52.1982246883 7.7198577500

H82 27.8102102970 51.4600205635 9.3152486810

H83 27.2496823772 54.4304770030 8.7460711120

H84 25.9959558866 53.2351611698 8.9611220923

H85 31.4844729653 52.0518918251 8.4602353237

H86 30.2611856337 51.6080410920 7.2630927246

H87 30.3198116541 50.7699459745 8.8224607822

H88 19.1275046513 59.2379047034 16.5037378059

H89 18.3680329637 59.1648648469 18.1021526813

H90 19.2600508293 57.7679963129 17.4869121412

H91 20.4801070731 60.5141226236 18.2166520745

H92 20.6068275059 59.0716671333 19.2081519149

H93 21.7349260113 56.8652037000 17.6683373348

H94 23.8667978041 56.7892385023 16.3071784397

H95 24.1804464013 66.8182894763 15.0625109702

H96 24.2500346351 65.3648223690 16.0682806143

H97 23.2011701727 65.4032765101 14.6400978546

H98 25.2374243664 65.5972805976 13.1533975510

H99 26.2764214693 65.5711054412 14.5674637039

H100 25.9069256748 63.4474347106 16.1858749703

H101 24.8540166604 63.5345415032 12.0219549164

H102 24.9063205842 61.0666114874 11.9513079753

H103 25.9970677000 60.9783040820 16.1206495729

H104 32.7345161595 55.7797457307 23.1755009972

H105 31.0183041005 55.5847071497 22.8190618570

H106 31.9219636891 56.9358953036 22.1117510335

H107 33.3653646396 55.2736230270 20.8430229978

H108 32.3956992918 53.9548670562 21.4743298343

H109 31.3860003595 56.2065878511 19.6576930196

H110 31.8022506766 54.5793944854 19.1276526857

H111 30.0001013907 53.6504761245 20.6567997276

H112 29.5069199450 55.2894153763 21.0434010790

H113 29.4485815384 54.0254255428 18.2437375374

H114 28.0621132522 54.2527856869 19.3066827386

H115 28.5446571452 56.6514966826 19.1850192578

H116 29.4251601031 62.3245560072 17.7668229337

H117 28.3513966016 62.3735438069 16.3593237235

H118 29.8925148286 61.5239961611 16.2728412915

H119 26.9559533011 60.8136529003 20.5245739912

H120 28.5429282956 61.4221822833 20.1354677117

H121 26.8722752577 63.2621682312 20.2418215450

H122 25.9210907006 62.5096537986 18.9536024259

H123 27.5319292694 63.1567392252 18.6095679061

H124 31.0295639695 59.2416872171 13.5511823625

H125 31.8262363854 57.9300063211 12.7154182530

H126 29.0270869662 59.0101202710 12.0182540708

H127 29.8499357609 57.6903042308 11.1647796354

H128 30.5110631179 59.3315367629 11.1041216083

H129 29.6181588206 59.7017102866 15.0929908766

H130 25.8821591980 59.0502465876 20.2339482095

H131 29.4466423471 53.3667455506 14.3022614589

H132 29.2438489328 50.9952491522 15.2281417047

H133 27.9777622727 51.1686176189 14.0150577311

H134 27.7440920869 50.0832174140 15.3904174278

H135 26.2764789765 50.0120171767 17.1134750756

H136 25.4616677035 51.0161924456 18.2948657892

H137 23.7959703479 50.0009123078 16.7758507248

H138 23.8185595411 51.7698030270 16.5224329238

H139 24.6490961573 53.0562609122 18.4996466631

H140 31.4436030147 55.7479865795 12.0398516887

H141 30.0819702395 54.6253259149 11.9984143691

H142 31.4215296631 54.3422025557 13.1050643949

H143 24.7261276815 57.8284654869 22.0252115048

H144 23.2850303110 56.8146521982 21.9696976207

H145 23.3533746494 58.3108826630 21.0323077882

H146 23.4865617958 53.4197480374 20.2042573876

H147 23.2661229971 54.5524917371 21.5108853345

H148 21.1082887613 54.0393787276 20.7002311285

H149 21.4358530536 55.7121286212 20.2110802311

H150 25.2822284542 49.2733175021 13.7820177114

H151 20.9792755784 52.8776489580 17.4803679054

O152 25.9580922248 56.7851025044 15.4822942628

H153 29.6641121580 56.3614536214 18.0407946223

H154 22.6754369185 60.8368491355 16.4790445742

H155 24.3281082802 59.1338615110 15.4992666960

H156 26.2834775733 57.6581975946 15.1909969426

H157 25.1750517102 59.1557108685 13.1863309848

H158 26.2845159874 56.1579741488 14.8155079318
